# Revisiting H‑Bond *v*. PT: The Role of Precursor and Successor Complexes in Intermolecular, Stepwise Proton-Coupled Electron Transfer

**DOI:** 10.1021/acsomega.5c06783

**Published:** 2025-10-21

**Authors:** Nikki Williams, Tanay Parnaik, Saptarshi Dutta, Joseph Bergen, Mauricio Cattaneo, Giovanny A. Parada

**Affiliations:** † Chemistry Department, 3280 The College of New Jersey, Ewing, New Jersey 08628, United States; ‡ Instituto de Química Física, Facultad de Bioquímica, Química y Farmacia, Universidad Nacional de Tucumán, 385667 INQUINOA (CONICET-UNT), Ayacucho 471 (4000), San Miguel de Tucumán, Argentina

## Abstract

Multiple mechanisms have been proposed to explain the electrochemical proton-coupled electron transfer (PCET) of arylenediamines upon weak base addition in aprotic media. Similar to quinone electrochemistry, endergonic deprotonation to form freely diffusing products is a central criterion used to exclude proton transfer. However, the second oxidation wave of arylenediamines shows large, chemically reversible *E*_1/2_ shifts—often exceeding hundreds of millivolts—upon base addition. To explain these effects, proposed mechanisms invoke strong H-bonding or H-bonding followed by nonconcerted PCET. Here, we elucidate a new mechanism using cyclic voltammetry of a new arylenediamine series where the p*K*_a_ is varied via π-electron spacers. A thermochemical analysis, based on equilibrium constants derived from biphasic *E*_1/2_ shifts observed from substoichiometric to excess base concentrations, supports a stepwise mechanism. This mechanism involves two sequential heterogeneous electron transfer steps (EE) followed by three homogeneous chemical steps (CCC), constituting an overall EECCC electrochemical mechanism. The CCC coupled equilibria correspond to exergonic H-bond precursor complex formation, thermoneutral proton transfer (PT), and endergonic successor complex dissociation. The energy well formed by the CCC coupled equilibria reconciles previous thermochemical analyses and provides a new explanation for chemically reversible electrochemical waves. Furthermore, a reactivity continuum between H-bonding and PT is demonstrated, which contrasts with the prevailing view, where either H-bonding or PT dominates the mechanism.

## Graphical abstract

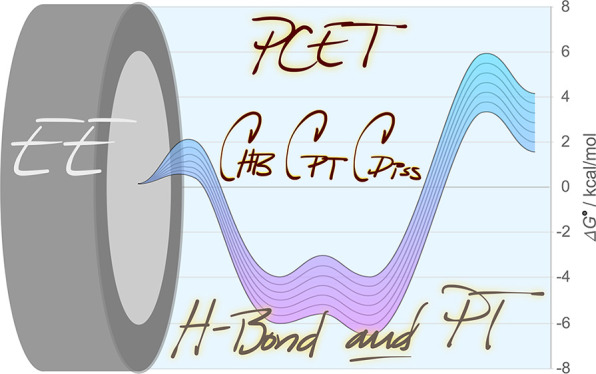

## Introduction

The coupled transfer of electrons and protons is a fundamental reaction of ground and excited states across numerous chemical and biological processes.^1–8^ Controlling such proton-coupled electron transfer (PCET) reactions is key to the development of solar fuels,^9,10^ as well as activation and synthesis of small molecules such as H_2_,^11^ N_2_ ^12^ O_2_,^13^ CO_2_,^14^ and of a wide range of organic functional groups.^8,15^ PCET mechanisms depend on (1) the energy of reactants, products, and intermediates, (2) the intrinsic barriers for elementary charge transfer steps: electron transfer (ET), proton transfer (PT), and concerted electron–proton transfer (CPET, where both the *e* ^–^ and H^+^ transfer in a single elementary step), and (3) the wave function overlaps between reactant and product states for ET, PT, and CPET.^4–6,16,17^ H-bonding affects most of these parameters because it can stabilize reactants, intermediates, and products, prealign the PT coordinate, change the proton donor–acceptor vibration frequency, and the shape of PT free energy profiles, thereby affecting PT wave function overlaps.^16,18^ In addition, for intermolecular reactions, the formation and dissociation of H-bonded precursor and successor complexes can significantly change the energy landscape and mechanism.^19–25^ Therefore, one of the primary challenges in PCET studies involves disentangling the structural and energetic contributions of H-bonding.

Quinone electrochemistry has been studied for decades to uncover the mechanistic effects of H-bonding on PCET reactivity.^26–31^ In aprotic media, quinone reduction potentials are controlled over wide ranges by varying the strength and concentration of H-bond donors.^32–47^ As the H-bond strength increases, so does the PT driving force.^32,48–57^ In a remarkable effort to separate the contributions of H-bonding and PT, Gupta and Linschitz mapped general zones of distinctive quinone electrochemical behavior in the presence of weak, intermediate, and strong H-bond donors.^32^ The protonation free energy to form freely diffusing products (Δ*G*_T_^°^) is the central criterion to rationalize differences in electrochemical behavior and formulate the mechanism for the different zones. The mechanistic proposals are based on the premise that either H-bonding or PT dominates following heterogeneous ET. Negligible PT is proposed for weak and intermediate strength H-bond donors because protonation to form freely diffusing products is endergonic, Q^2–^+ HA ⇌ QH^–^ + A^–^, Δ*G*_T_^°^ = – *RT*ln10 × Δp*K*_a_. The proposed mechanism is therefore exclusively based on H-bond stabilization. PT is proposed only for strong H-bond donors. This view of H-bond *v*. PT—neglecting one effect from the mechanism—has provided a paradigm for thinking about stepwise, intermolecular PCET even beyond quinone electrochemistry.^26^ However, a reactivity continuum is likely because the driving forces for H-bonding and PT increase concurrently.

In contrast to quinone electrochemistry, the analogous electrochemical oxidation of arylenediamines in aprotic media with weak H-bond acceptors cannot be accounted for exclusively by H-bonding (Scheme 1).^58–61^ The large *E*_1/2_ shifts of the second oxidation suggest an unattainable very strong H-bond, unless additional charge transfer steps are involved. Large *E*_1/2_ shifts of the second reduction in quinones are explained by the sequential formation of H-bonded adducts {Q^2–^···(H-A)_*n*_} with various stoichiometries (1:*n* from 1:1 to 1:6) at equilibrium.^40–47^ A similar scenario is not possible for arylenediamines, because their H-bond stoichiometry is limited to one H-bond per nitrogen. Yet, arylenediamines and quinones show comparable *E*_1/2_ shifts of their second oxidation/reduction. Smith and co-workers proposed that a PCET sequence couples with H-bonding to account for the large *E*_1/2_ shifts of the second oxidation.^58^ Specifically, they propose a mechanism with pre-equilibrium H-bonding of the monocation (A^•+^-H_2_) followed by a stepwise PCET sequence within the H-bonded adduct: {A^•+^-H_2_···B} – *e* ^–^ ⇌ {A^+^-H···HB^+^}. The stepwise PCET sequence is termed *nonconcerted* PCET, accounting for the lack of kinetic isotope effect, even though KIE interpretations depend on multiple factors.^62^ By coupling the second oxidation with PT, the standard reduction potential for the PCET step must fall between the potentials of the non-H-bonded protonated (A^2+^-H_2_/A^•+^-H_2_) and deprotonated (A^+^-H/A^•^-H) redox couples, which are expected to be apart by hundreds of millivolts.^7^ The mechanism derives from Smith’s PCET Wedge scheme, where H-bonding is incorporated into PCET square schemes.^63,64^ In contrast to analogous quinone reactivity, both H-bonding and the *nonconcerted* PCET are proposed to be necessary to account for the electrochemical behavior of arylenediamines, even at a low driving force using weak H-bond acceptors.

**Scheme 1:**
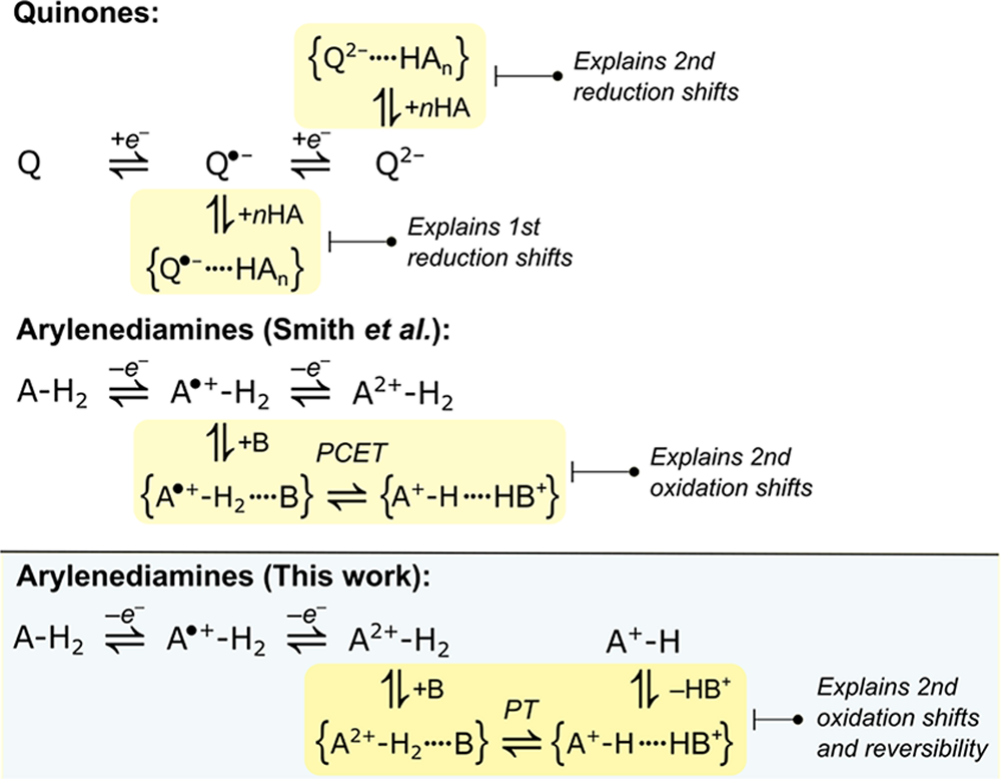
H-Bonding and Charge Transfer Steps Are Proposed to Explain the Redox Potential Shifts of Quinones and Arylenediamines in Aprotic Media upon the Addition of Weak H-Bond Donors/Acceptors^a^

a The Mechanism by Smith and co-workers is from ref 58.

Here, we provide evidence supporting an alternative mechanism for the oxidation of arylenediamines, accounting for the observed large *E*_1/2_ shifts of the second oxidation. Three arylenediamines were studied by cyclic voltammetry (CV) in the presence of substoichiometric and excess weak H-bond acceptors: 3,5-dichloro-pyridine (3,5-diCl-py), pyrazine (pyrz), and 4-cyanopyridine (4-CN-py). The arylenediamines feature π-spacers of different lengths from phenyl, naphthyl to diphenyl (Scheme 2). The series is designed to control the extent of electronic coupling between the two (1*e* ^–^/1H^+^) redox centers, which in this study allows us to modulate the electrostatic repulsion between the dications (A^2+^-H_2_), thereby systematically varying the splitting of redox potentials and Δp*K*_a_ between monocations and dications.^65^ Via the analysis of thermochemical data summarized on free energy profiles, we demonstrate that the formation *and* dissociation of precursor and successor complexes are both critical in the mechanistic analysis. Moreover, we show that H-bonding and PT operate on a continuum, even when forming freely diffusing PT products is endergonic.

**Scheme 2:**
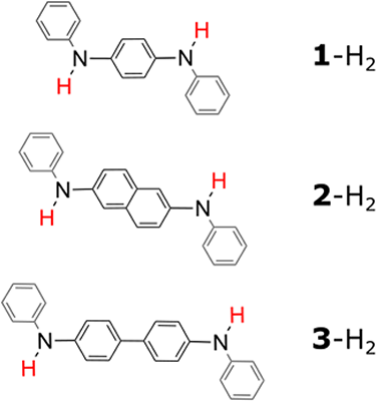
Studied Arylenediamines with Varying π-Electron Spacers

## Results and Discussion

For an electrochemical step followed by a first-order homogeneous chemical step (EC mechanism), a reversible Nernstian wave is expected when the rate of the chemical step is larger than the diffusion flux.^66–68^ The reversible Nernstian wave is centered around the formal potential, as the reduced and oxidized species readily equilibrate with the homogeneous chemical step. The equilibrium constant can be calculated from formal potentials by

$$E^{{}^{\circ}\prime }=E^{{}^{\circ}}+\frac{RT}{F}\ln(1+K)$$ 1

where *E*° is the formal potential of the electrochemical step, *K* is the equilibrium constant of the homogeneous chemical step, and *E*°′ is the formal potential of the coupled electrochemical step. For electroactive H-bond acceptors/donors following an EC mechanism, the equilibrium constant *K*_HB_ can be calculated from *E*_1/2_ shifts varying the concentration of H-bonding donor/acceptor.^32–35,40–47^

### Monocation vs Dication H-Bonding

CVs of the arylenediamines **1**-H_2_, **2**-H_2_, and **3**-H_2_ (collectively termed A-H_2_ here) in the presence of weak H-bond acceptors (bases, B = 3,5-diCl-py, pyrz, or 4-CN-py) are shown in Figure 1. In the absence of base, the difference between the first and second oxidation *E*_1/2_ values (=*E*_1/2_^(2)^ – *E*_1/2_^(1)^) decreases as the π-spacers length increases (**1**-H_2_ < **2**-H_2_ < **3**-H_2_) as expected for electronically coupled redox centers (Table 1).^65^ The first oxidation corresponds to the formation of the monocation (A^•+^-H_2_) and the second one to the formation of the dication (A^2+^-H_2_). The A^•+^-H_2_ shows the expected intervalence charge transfer bands in the near-IR (Figure S7). Titration with base causes cathodic shifts on *E*_1/2_^(2)^ while *E*_1/2_^(1)^ remains constant. Additional features in the CVs of **2**-H_2_ and the apparent merging of *E*_1/2_^(2)^ and *E*_1/2_^(1)^ for **3**-H_2_ are discussed in the SI.

**Figure 1:**
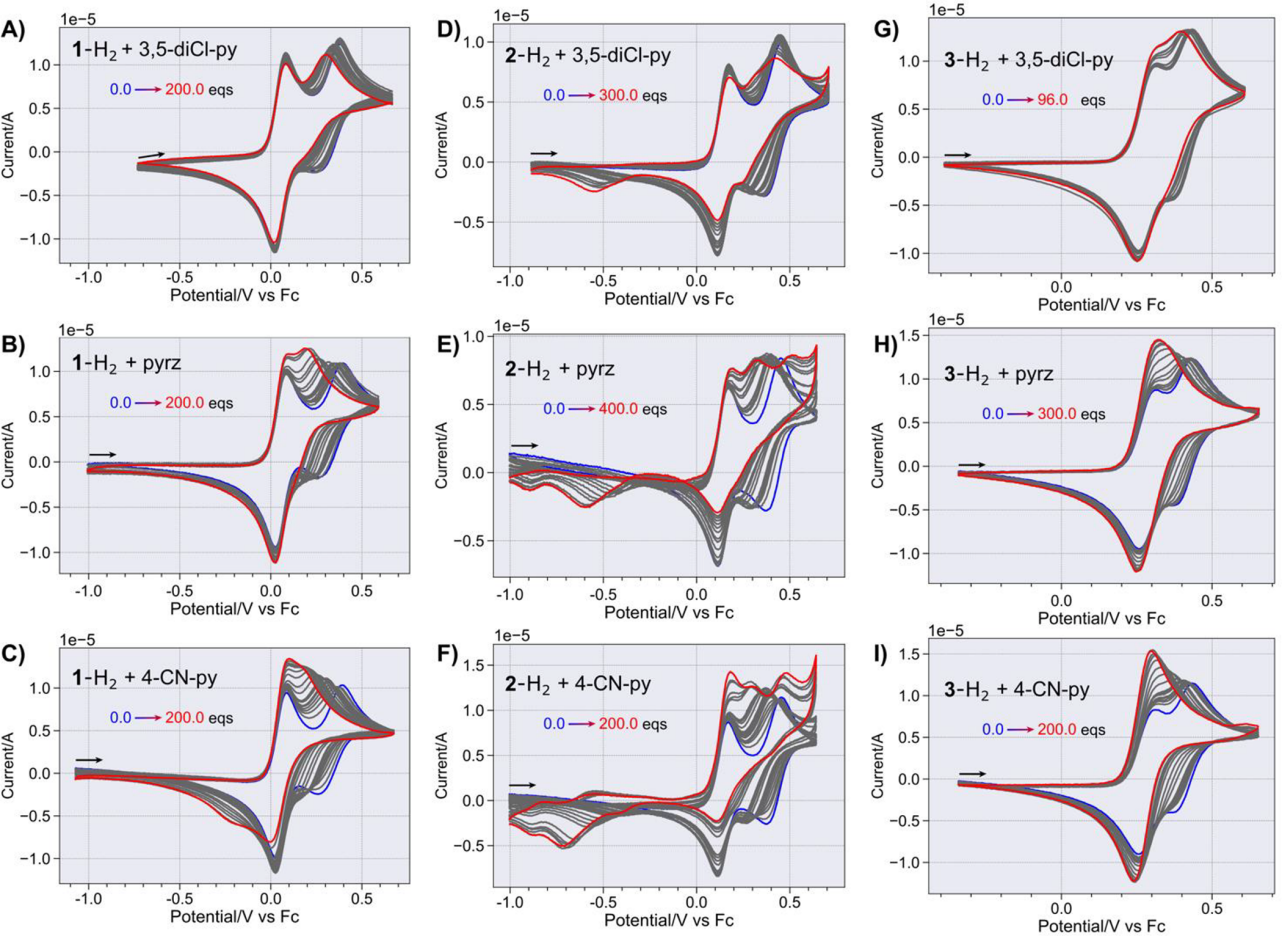
Background-subtracted CVs for arylenediamines **1**-H_2_, **2**-H_2_ and **3**-H_2_ (0.3–0.5 mM) in 0.1 M NBu_4_PF_6_ in CH_3_CN with and without added 3,5-diCl-py, pyrz, and 4-CN-py at 200 mV/s. Without added base (in blue) and with successive added base from substoichiometric to a large excess between 100 and 400 equiv (in red). Panels A–C: **1**-H_2_ with 3,5-diCl-py, pyrz and 4-CN-py, respectively; panels D–F: **2**-H_2_ with 3,5-diCl-py, pyrz and 4-CN-py, respectively; and panels G–I: **3**-H_2_ with 3,5-diCl-py, pyrz and 4-CN-py, respectively.

**Table 1:** Electrochemical Parameters of the Oxidation of Arylenediamines in the Presence of Bases

	*E*_1/2_^(1)^ ^a^	*E*_1/2_^(2)^ ^a^	base	*K*_HB,2_	*n* (0–2 eqs)	*K*_obs_	*n* (>2 eqs)
**1**-H_2_	0.052	0.423	3,5-diCl-py	7.8(7) ×10^3^	1.0(2)	6.2(8) × 10^3^	1.0(1)
			pyrz	1.3(2) × 10^4^	1.3(5)	6.0(7) × 10^4^	1.3(1)
			4-CN-py	2.05(9) × 10^8^	1.9(1)	2.0(1) × 10^5^	1.3(2)
**2**-H_2_	0.142	0.422	3,5-diCl-py	8.3(4) × 10^2^	0.81(5)	4.2(8) × 10^2^	0.70(7)
			pyrz	3.7(1) × 10^3^	1.24(6)	3.2(6) × 10^3^	0.9(1)
			4-CN-py	3.0(2) × 10^7^	1.9(2)	1.0(2) × 10^4^	0.8(1)
**3**-H_2_	0.280	0.400	3,5-diCl-py	3.5(4) × 10^2^	0.7(2)	1.1(1) × 10^2^	0.7(1)
			pyrz	7.0(6) × 10^2^	0.81(6)	8.5(4) × 10^2^	1.10(7)
			4-CN-py	5.0(1) × 10^3^	1.17(5)	1.0(1) × 10^4^	1.29(8)

a Potential in volts vs Fc^+/0^ in CH_3_CN. Values of equilibrium constants and stoichiometric coefficients are calculated from best linear fits according to eq 6 before 2 equiv of base, and to eq 11 after 2 equiv of base, where *K*_obs_= *K*_HB_·*K*_PT_. Uncertainties from linear fits are used to estimate the errors, calculated as confidence intervals, in parentheses, at a 95% confidence limit.

The cathodic shifts on *E*_1/2_^(2)^ while *E*_1/2_^(1)^ remains constant are strong evidence that H-bonding is large for A^2+^-H_2_ but negligible for A^•+^-H_2_ (*K*_HB,2_ ≫ 1 and *K*_HB,1_ ≈ 0, where *K*_HB,1_ and *K*_HB,2_ are the H-bond equilibrium constants for A^•+^-H_2_ and A^2+^-H_2_, respectively). Related arylenediamines show similar behavior.^58–61,63,64,69^ For comparison, quinones can show *E*_1/2_^(1)^ shifts, though smaller than those of *E*_1/2_^(2)^ as *K*_HB,1_ becomes non-negligible.^32–35,40–47^ The relation *K*_HB,2_ > *K*_HB,1_ results from the larger acidity of A^2+^-H_2_ vs A^•+^-H_2_ due to charge accumulation and repulsion in A^2+^-H_2_.^65^ In the A-H_2_ series, this correlation is confirmed by a Hammett-type analysis; see Section 5 in the SI for details. These redox and H-bonding reactions correspond to an EEC_HB_ electrochemical mechanism, eqs 2–5. In eq 5, *n* = 1 or 2 because there are two symmetrical H-bond donors per A^2+^-H_2_. The equilibrium constants *K*_HB,2_ and *n* can be calculated from *E*_1/2_^(2)^ shifts varying the concentration of B, according to eq 6, where Δ*E*_1/2_ ≈ *E*_1/2_^(2)^ – *E*_1/2_^(2)′^ (= *E*_2_^°^ – *E*_2_^°′^).

$$\begin{array}{cc} A^{•+}\text{‐}H_{2}+e^{-}⇌A-H_{2} & E_{1}^{{}^{\circ}} \end{array}$$ 2

$$\begin{array}{cc} A^{•+}\text{‐}H_{2}+B⇌\{A^{•+}\text{‐}H_{2}\cdot \cdot \cdot B\} & K_{\mathrm{HB},1}\;\underset{\_}{\mathrm{negligible}} \end{array}$$ 3

$$\begin{array}{cc} A^{2+}\text{‐}H_{2}+e^{-}⇌A^{•+}\text{‐}H_{2} & E_{2}^{{}^{\circ}} \end{array}$$ 4

$$\begin{array}{cc} A^{2+}\text{‐}H_{2}+nB⇌\{A^{2+}\text{‐}H_{2}\cdot \cdot \cdot B_{n}\} & K_{\mathrm{HB},2} \end{array}$$ 5

$${\Delta E}_{1/2}=\frac{RT}{F}\ln\left(1+K_{\mathrm{HB},2}{\left[B\right]}^{n}\right)$$ 6

Smith and co-workers^58^ propose that the monocation A^•+^-H_2_ forms a H-bonded adduct that subsequently undergoes a PCET step, resulting in an overall EC_HB_-PCET mechanism, eqs 2, 7, and 8. This disregards the fact that *E*_1/2_^(1)^ does not shift and wrongly assumes that *K*_HB,1_ is not negligible.

$$\begin{array}{cc} A^{•+}\text{‐}H_{2}+B⇌\{A^{•+}\text{‐}H_{2}\cdot \cdot \cdot B\} & K_{\mathrm{HB},1} \end{array}(\mathrm{assumed}\;\mathrm{non}‐\mathrm{negligible})$$ 7

$$\begin{array}{cc} \left\{A^{•+}\text{‐}H_{2}\cdot \cdot \cdot B\}-e^{-}⇌\{A^{+}\text{‐}H_{2}\cdot \cdot \cdot {\mathrm{BH}}^{+}\right\} & E_{2}^{{}^{\circ}\prime }(\mathrm{PCET}) \end{array}$$ 8

Moreover, the Smith mechanism is unlikely to outcompete the EEC_HB_ electrochemical mechanism outlined above. Let us assume that *K*_HB,1_ is not negligible and {A^•+^-H_2_···B} forms. Then, for EC_HB_-PCET to outcompete EEC_HB_, the second heterogeneous ET must be faster for {A^•+^-H_2_···B} than for A^•+^-H_2_. This scenario could occur only if for {A^•+^-H_2_···B} the heterogeneous ET is coupled to PT. Without a PT-coupled step, a non-negligible but at best very weak H-bond is unlikely to increase the heterogeneous ET rates significantly. However, the difference in heterogeneous ET rates between EEC_HB_ and EC_HB_-PCET is expected to be very large. Both mechanisms require a H-bond step either before or after the second heterogeneous ET, and therefore, the difference in heterogeneous ET rates should be at least as large to outcompete the favored H-bond formation of A^2+^-H_2_ over A^•+^-H_2_. Because the difference in acidity of **1** ^•+^-H_2_ vs **1** ^2+^-H_2_ is large, Δp*K*_a_ ∼ 9 (vide infra); for EC_HB_-PCET to outcompete EEC_HB_, the relative heterogeneous ET rates must be at least 9 orders of magnitude faster for {**1** ^•+^-H_2_···B} vs **1** ^•+^-H_2_, assuming similar transfer coefficients.^68^ Even assuming a very slow heterogeneous ET to **1** ^•+^-H_2_ and highly exergonic PCET, a 9 orders of magnitude difference in rates is unrealistic. In other words, the EC_HB_-PCET proposed by Smith and co-workers^58^ implies an unlikely, highly exergonic and fast PCET step, even assuming non-negligible *K*_HB,1_.

### Stoichiometric Dependence

For every A-H_2_ and base combination used, the *E*_1/2_^(2)^ shifts by different extents for substoichiometric and excess amounts of base, see Figures S9–S20 and Table 1. This biphasic behavior is shown on the fits according to the EEC_HB_ mechanism as different slopes before and after 1 equiv of added base per acid site (2 equiv per A^2+^-H_2_). Rearranging eq 6 allows us to construct plots of exp­(*f*Δ*E*_1/2_) – 1 vs [*B*], where *f* = *F*/*RT* and the slope equals *K*, whereas in logarithmic plots, log_10_(exp (*f*Δ*E*_1/2_) – 1) vs log_10_([*B*]), the slope equals *n* (Figures S9–S20). *K* and *n* values from these analyses are reported in Table 1 with uncertainties at the 95% confidence level in parentheses. Differences in *K* values between substoichiometric and excess base are statistically significant with a 95% confidence level for every A-H_2_ and base combination according to the *t*-test.

For substoichiometric base amounts, *K* values are assigned to *K*_HB,2_ consistent with the arguments favoring H-bonding on A^2+^-H_2_ vs A^•+^-H_2_. *K*_HB,2_ spams 6 orders of magnitude across the A-H_2_ and base combinations (Table 1). For each A-H_2_, *K*_HB,2_ increases as the basicity of the base increases: 3,5-diCl-py < pyrz <4-CN-py; the p*K*_a_ values of their iminiums in CH_3_CN are 3,5-diCl-pyH^+^ (∼5.3) < pyrzH^+^ (7.74) < 4-CN-pyH^+^ (8.50) in CH_3_CN. For each base, *K*_HB,2_ follows the trend **1** ^2+^-H_2_ > **2** ^2+^-H_2_ > **3** ^2+^-H_2_ as the p*K*_a_ values of the dications decrease with increased charge repulsion as the π-spacer becomes shorter (Table 2). The observed trends with the p*K*_a_ values for A^2+^-H_2_ and bases are consistent with the assignment of *K*_HB,2_.

**Table 2:** Thermochemical Parameters for H-Bonded Precursor Complex Formation, Proton Transfer, and H-Bonded Successor Complex Dissociation

	p*K*_a_^(1)^	p*K*_a_^(2)^	base	Δ*G*_T_^°^ ^c^	Δ*G*_HB,2_^°^ ^d^	Δ*G*_PT_^°^ ^d^	Δ*G*_diss_^°^ ^e^
**1**-H_2_	15.2^a^	6.70^a^	3,5-diCl-py	2.0	–5.33	0.14	7.1
			Pyrz	–1.4	–5.64	–0.91	5.1
			4-CN-py	–2.5	–11.39	–1.57^f^	4.8
**2**-H_2_	16.2^b^	9.6^b^	3,5-diCl-py	6.0	–4.00	0.41	9.5
			Pyrz	2.6	–4.89	0.09	7.3
			4-CN-py	1.5	–10.24	–0.36^f^	7.0
**3**-H_2_	15.4^b^	10.7^b^	3,5-diCl-py	7.4	–3.49	0.69	10.2
			Pyrz	4.1	–3.90	–0.12	8.1
			4-CN-py	3.0	–5.07	–0.41	8.5

a p*K*_a_ from thermochemical cycles using acidity equilibria and bond dissociation free energies for 1^2+^-H_2_.

b p*K*_a_ from B3LYP+6–311++G­(2d,2p)/SMD DFT calculations using a solvent continuum of CH_3_CN.

c Calculated using the p*K*_a_ of the dications and pyridiniums in CH_3_CN.

d Calculated in kcal/mol from experimental *K*_HB,2_ and *K*_obs_= *K*_HB,2_·*K*_PT_.

e Calculated in kcal/mol from Δ*G*°_diss_ = Δ*G*°_T_ – Δ*G*°_HB,2_ – Δ*G*°_PT_.

f Calculated in kcal/mol using *K*_PT_ = *K*_obs_/(*K*_HB,2_)^1/2^ because the experimental *K*_HB,2_ corresponds to the H-bonding of 2 equiv of base.

The *n* values show an A^2+^-H_2_ to base stoichiometry that is 1:1 (*n* = 1) for all A-H_2_ and bases except for **1** ^2+^-H_2_ and **2** ^2+^-H_2_ with 4-CN-py, for which the stoichiometry is 1:2 (*n* = 2), see Table 1. The 1:2 stoichiometry suggests that each aminyl radical cation (Ar)_2_N^•+^–H is H-bonded to one base in symmetric A^2+^-H_2_. The combinations of **1** ^2+^-H_2_ and **2** ^2+^-H_2_ with 4-CN-py show clear quadratic exp­(*f*Δ*E*_1/2_) – 1 vs [*B*] plots consistent with *n* = 2 in the logarithmic plots (see Figures S12 and S16). The 1:2 stoichiometry is only observed for the strongest acid/base pairs with the largest Δp*K*_a_ and *K*_HB,2_ as they have sufficient driving force to overcome the entropic penalty for assembling a higher-order complex and the electrostatic penalty for binding a base to a complex where the dication’s charge density has been attenuated by the formation of the first H-bond. Only the combinations with the largest *K*_HB,2_ have sufficiently large H-bond formation enthalpy to overcome the entropic and electrostatic penalties to reach the 1:2 stoichiometry. This stoichiometric dependence is only observed for substoichiometric base amounts, consistent with the assignment of *K*_HB,2_.

According to the EC_HB_-PCET mechanism, the base concentration-dependent step is the formation of {A^•+^-H_2_ ···B}. Even if {A^•+^-H_2_···B} is assumed to be a stable intermediate, it undergoes unimolecular, exergonic, and fast PCET. On the other hand, the observed biphasic behavior and stoichiometry suggest a base concentration-dependent step that forms a discrete, thermodynamically stable complex that readily accumulates and reversibly equilibrates at the electrode’s surface. The unimolecular, exergonic, and fast PCET is also inconsistent with the 1:2 stoichiometry attained for the combinations with the largest driving force.

### Proton Transfer and Successor Complex Dissociation

So far, we have considered how the larger acidity of A^2+^-H_2_ vs A^•+^-H_2_, lack of *E*_1/2_^(1)^ shifts, and base concentration-dependence support the assignment of substoichiometric *K* values to *K*_HB,2_ in support of the EEC_HB_ mechanism. However, the *E*_1/2_^(2)^ values continue shifting as more base is added beyond one equivalent per acidic site in A^2+^-H_2_. The *E*_1/2_^(2)^ shifts occur with a different *K* value compared to substoichiometric amounts (biphasic behavior). This indicates that additional equilibrium processes occur following EEC_HB_. However, subsequent H-bonding is unlikely due to the H-bond saturation of 1:2 adducts and the difficulty in overcoming the entropic and electrostatic penalties of forming them from 1:1 adducts as Δp*K*_a_ decreases. Consequently, we hypothesize that the H-bond adducts {A^2+^-H_2_···B_*n*_}, whether *n* = 1 or 2, are subsequently deprotonated, with PT occurring at equilibrium between H-bonded precursor and successor complexes (eq 9). The H-bonded successor complex can further dissociate to yield the freely diffusing, doubly oxidized, deprotonated arylenediamines and conjugated acids (eq 10). Extension of the coupled equilibria expression derived from eq 1 to account for coupled PT results in eq 11, whereas extension to additionally account for the successor complex dissociation results in eq 12.^34^

$$\begin{array}{cc} \left\{A^{2+}\text{‐}H_{2}\cdot \cdot \cdot B_{n}\}⇌\{A^{+}\text{‐}H_{2}\cdot \cdot \cdot B_{n}H^{+}\right\} & K_{\mathrm{PT}} \end{array}$$ 9

$$\begin{array}{cc} \{A^{+}\text{‐}H\cdot \cdot \cdot B_{n}H^{+}\}⇌\{A^{+}\text{‐}H\cdot \cdot \cdot B_{n-1}\}+{\mathrm{HB}}^{+} & K_{\mathrm{diss}} \end{array}$$ 10

$${\Delta E}_{1/2}=\frac{RT}{F}\ln\left(1+K_{\mathrm{HB},2}K_{\mathrm{PT}}{\left[B\right]}^{n}\right)$$ 11

$${\Delta E}_{1/2}=\frac{RT}{F}\ln\left(1+\frac{K_{\mathrm{HB},2}K_{\mathrm{PT}}K_{\mathrm{diss}}}{2}{\left[B\right]}^{n}\right)$$ 12

The equilibrium constants after 2 equiv, denoted *K*_obs_ in Table 1, show the same trends described for *K*_HB,2_, i.e., *K*_obs_ increases as Δp*K*_a_ between A^2+^-H_2_ and bases increases. To test whether *K*_obs_ corresponds to that of eq 11 or 12, we use the overall PT free energy (Δ*G*_T_^°^); that is, the PT free energy between freely diffusing reagents and products calculated from the p*K*_a_ values of the A^2+^-H_2_ and base (eqs 13–15). Δ*G*_T_^°^ totals the free energy for the sequence: H-bonded precursor complex formation, PT, and successor complex dissociation by Hess’s law (eq 16).

$$\begin{array}{cc} {\mathrm{BH}}^{+}⇌H^{+}+B & K_{a,\mathrm{base}} \end{array}$$ 13

$$\begin{array}{cc} A^{2+}\text{‐}H_{2}⇌A^{+}\text{‐}H+H^{+} & K_{a,\mathrm{dication}} \end{array}$$ 14

$$\begin{array}{cc} A^{2+}\text{‐}H_{2}+B⇌A^{+}\text{‐}H+{\mathrm{BH}}^{+} & \Delta G_{T}^{{}^{\circ}}=-RT\ln\left(\frac{K_{a,\mathrm{dication}}}{K_{a,\mathrm{base}}}\right) \end{array}$$ 15

$$\Delta G_{T}^{{}^{\circ}}=\Delta G_{\mathrm{HB},2}^{{}^{\circ}}+\Delta G_{\mathrm{PT}}^{{}^{\circ}}+\Delta G_{\mathrm{diss}}^{{}^{\circ}}$$ 16

The p*K*_a_ values for A^2+^-H_2_ and the bases in CH_3_CN were taken from experimental values in the literature or calculated from thermochemical cycles from experimental values in other solvents or calculated by DFT with benchmarking using experimental values (see the SI). The p*K*_a_ for **1** ^2+^-H_2_ in CH_3_CN was calculated using thermochemical cycles^6^ with data reported in DMSO.^70,71^ The p*K*_a_ for **2** ^2+^-H_2_ and **3** ^2+^-H_2_ were calculated from DFT using a continuum solvent of CH_3_CN and benchmarking vs the calculated data for **1** ^2+^-H_2_. The calculated p*K*_a_ values reproduce the expected trend with **1** ^2+^-H_2_ > **2** ^2+^-H_2_ > **3** ^2+^-H_2_ due to larger charge repulsion as the π-spacers become shorter (Table 2). The p*K*_a_ for 3,5-diCl-py was calculated from DFT using a continuum solvent of CH_3_CN and benchmarking vs experimental data for other pyridines, including pyrz and 4-CN-py. The calculated p*K*_a_ trend is 3,5-diCl-pyH^+^ < pyrzH^+^ < 4-CN-pyH^+^. The calculated Δ*G*_T_^°^ values follow the expected trend based on the Δp*K*_a_ values between each A^2+^-H_2_ and base (Table 2).

An energy balance reveals the only possible assignment for *K*_obs_. Deprotonations to yield free diffusing reagents are, in general, endergonic, Δ*G*_*T*_^°^ > 0; only the most acidic **1** ^2+^-H_2_ shows exergonic deprotonations with pyrz and 4-CN-py (Table 2). On the other hand, formation of the H-bonded precursor complexes is exergonic, *K*_HB,2_ > 1 (Table 1 and Δ*G*_HB,2_^°^ < 0 in Table 2). *K*_obs_ indicate overall exergonic equilibria following EEC_HB_, *K*_obs_ > 1 (Table 1). Given these thermochemical values, the only *K*_obs_ expression that can simultaneously satisfy endergonic Δ*G*_T_^°^, implying *K*_T_ = *K*_HB,2_ · *K*_PT_ · *K*_diss_ < 1, as well as exergonic Δ*G*_HB,2_^°^ < 0 and *K*_obs_ > 1, corresponds to *K*_obs_ = *K*_HB,2_ · *K*_PT_. For comparison, assuming *K*_obs_ = *K*_HB,2_ · *K*_PT_ · *K*_diss_/2 (i.e., *K*_obs_ = *K*_T_/2) makes the observed *K*_obs_ > 1 and calculated Δ*G*_T_^°^ > 0 (*K*_T_ < 1) values contradictory.

Having assigned *K*_obs_, we calculate Δ*G*_PT_^°^ and Δ*G*_diss_^°^ (Table 2). With Δ*G*_HB,2_^°^, Δ*G*_PT_^°^, and Δ*G*_diss_^°^ in hand, we complete free energy profiles for the entire sequence of homogeneous reactions: H-bond precursor complex formation, PT, and H-bonded successor complex dissociation (Figure 2). Δ*G*_PT_^°^ is close to thermoneutral but varies from slightly endergonic to slightly exergonic as the Δp*K*_a_ between A^2+^-H_2_ and base increases (Table 2 and Figure 2). Thermoneutral PT suggests a symmetric PT energy profile.^72^ Dissociation of the successor complexes is endergonic, Δ*G*_diss_^°^ > 0, by several kcal/mol, and varies significantly across the series with a trend opposite to that of Δp*K*_a_ between A^2+^-H_2_ and bases: the more exergonic Δ*G*_HB,2_^°^ and Δ*G*_T_^°^, the least endergonic Δ*G*_diss_^°^. This is consistent with the expectation that the more exergonic Δ*G*_HB,2_^°^ and Δ*G*_T_^°^, the easier free diffusing deprotonation products can be formed.

**Figure 2:**
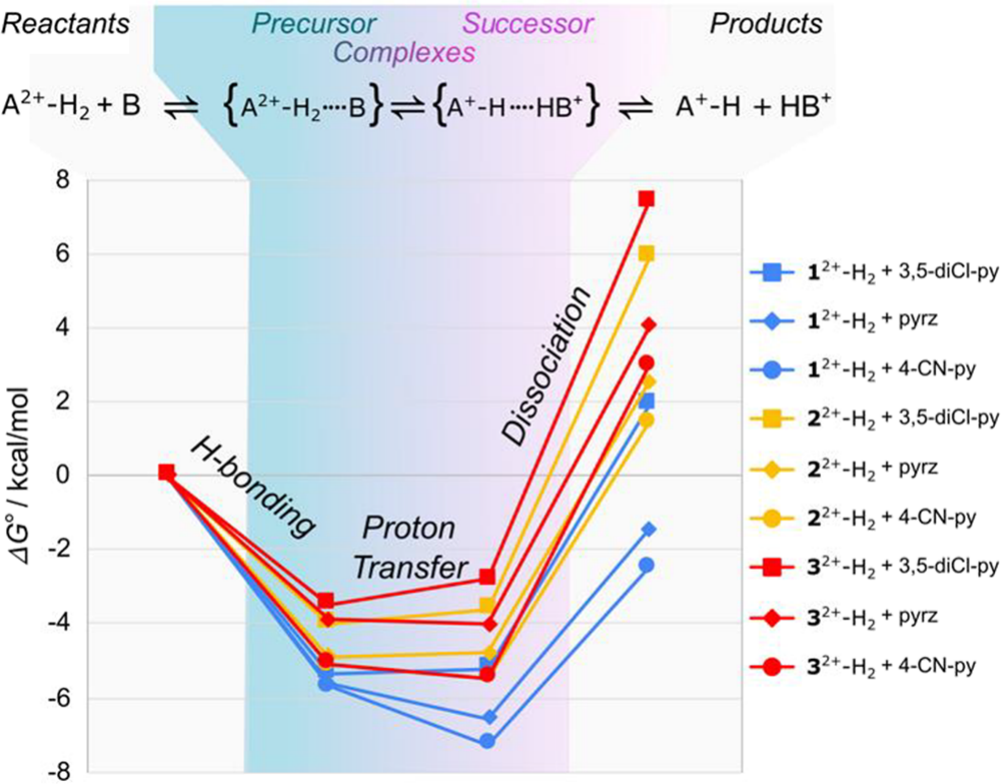
Gibbs free energy profiles for H-bonding formation of the precursor complex, proton transfer, and dissociation of H-bonded successor complex. Gibbs free energy profiles for 1^2+^-H_2_ (in blue), 2^2+^-H_2_ (in yellow), and 3^2+^-H_2_ (in red) with the pyridines: 3,5-diCl-py (■), pyrz (◆), 4-CN-py (●). The figure is from the data in Table 2.

A new reductive wave around −0.25 V vs Fc for **1**-H_2_ with 4-CN-py, assigned to the deprotonated **1** ^+^-H/**1** ^•^-H couple, is direct evidence of successor complex dissociation (Figure 1C). The wave is observed for **1**-H_2_ with 4-CN-py because this combination has the least endergonic Δ*G*_diss_^°^, see Table 2 and Figure 2. The assignment of the new reductive wave with potential *E*_4_^°^ is similar to that by Smith and co-workers,^58^ as the **1** ^+^-H/**1** ^•^-H couple is expected to have a potential more cathodic than *E*_1_^°^ (Scheme 3A).^6,7^ A small *E*_4_^°^ cathodic shift is observed at 200 equiv of 4-CN-py as the scan rate increases (Figure S21 panel G). This could be indicative of coupled steps in solution, beginning with the H-bonding of a conjugated acid in solution. At 0.2 V/s, the dissociation is only observed if the base concentration is larger than 100 equiv (Figure 1C). Observing the dissociation only for the combination with the least endergonic Δ*G*_diss_^°^ implies that for all other combinations, the reassociation of the *successor* complex is too exergonic (and fast). For the **1**-H_2_ with 4-CN-py combination, the reassociation can be outcompeted at faster scan rates (Figure S21), even for base concentrations lower than 100 equiv.

**Scheme 3:**
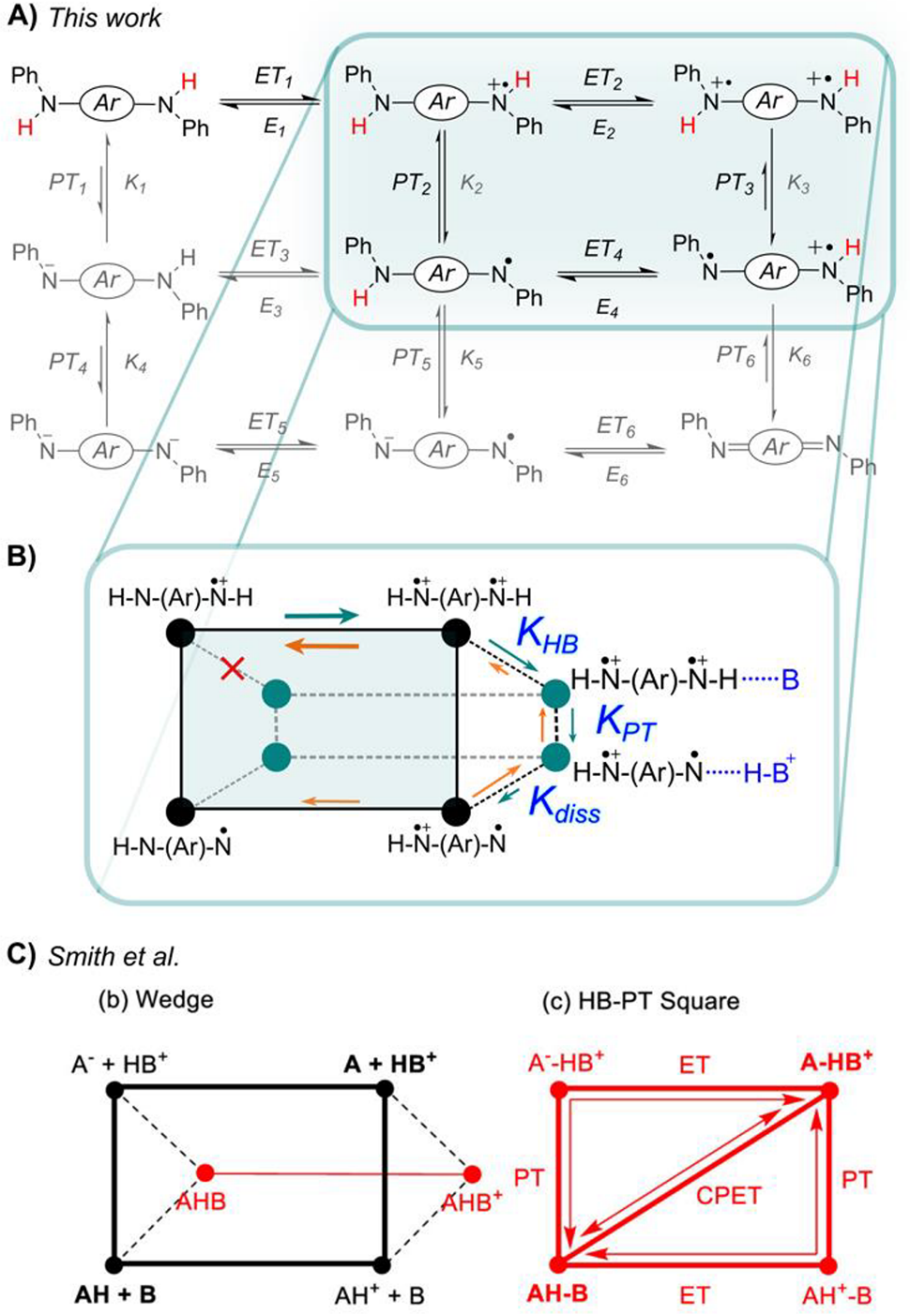
Square Schemes for 2*e* ^–^/2H^+^ Transfer of Arylenediamines and Proposed Wedge-like Schemes.^a^

a (A) 2*e* ^–^/2H^+^ square schemes excluding the formation and dissociation of precursor and sucessors complexes. Ar = aryl, all other abbreviations are as defined in the text. (B) Proposed wedge-like scheme for the stepwise ET-PT with H-bond precursor complex formation and H-bond successor complex dissociation. The wedge-like scheme corresponds to the expansion of the top-right quadrant of the 2*e* ^–^/2H^+^ square scheme to account for the formation and dissociation of H-bonded precursor and successor complexes. The red (X) represents the unlikely forward path via H-bonding of the mono-cation. (C) Wedge scheme for PCET by Smith and co-workers. Adapted in part with permission from ref 58. Copyright (2019) American Chemical Society. PCET reactants are in the bottom left corner of each panel. The square scheme in panel (C) corresponds to the wedged reaction AHB →AHB^+^ (in red) in the Wedge scheme in panel (B).

Homoconjugation between pyridiniums and pyridines, HB^+^ + B ⇌ {B^+^–H···B}, can contribute as an additional base concentration-dependent step driving the equilibria forward, but the effect is expected to be small (*K*_BHB_ ≈ 10–20 in CH_3_CN).^73^

### EEC_HB_C_PT_C_Diss_ Mechanism and Its Implications

The overall electrochemical mechanism is designated as EEC_HB_C_PT_C_Diss_. The dication, A^2+^-H_2_, is formed after two sequential oxidations. Then, a sequence of coupled reactions occurs in solution: (1) exergonic H-bonding to form a PT precursor complex, (2) thermoneutral PT, and (3) endergonic dissociation of the successor complex. The C_HB_C_PT_C_Diss_ sequence forms an energy well for every combination (Figure 2). The proposed mechanism is consistent with key experimental observations: lack of *E*_1/2_^(1)^ shifts and base concentration-dependence of large *E*_1/2_^(2)^ shifts with distinctive shift magnitudes before and after 1 equivalence of added base. Initial H-bonding follows from the fact that A^2+^-H_2_ is more acidic than A^•+^-H_2_. The interpretation of *E*_1/2_^(2)^ shifts in excess base follows from the stoichiometric dependence, indicating that initial H-bonding occurs before 2 equiv per A^2+^-H_2_. The coupled equilibria following H-bonding, assigned to PT and dissociation of the successor complex, are decoded from *K*_obs_ and a thermochemical analysis. Gibbs free energy profiles show that the coupled C_HB_C_PT_C_Diss_ equilibria follow the expected trends based on the Δp*K*_a_ between A^2+^-H_2_ and the base. Together, the thermochemical arguments and observed dissociation of the successor complex at high base concentrations or fast scan rates validate the key hypothesis of the mechanism: PT occurring at equilibrium between H-bonded precursor and successor complexes.

A wedge-like scheme for the EEC_HB_C_PT_C_Diss_ is shown in Scheme 3B. It has a wedge-like shape only for graphical convenience, as it could also form a cube. Nonetheless, it is inspired by the wedge scheme proposed by Smith and co-workers (Scheme 3C), whose merit is to integrate the role of H-bonding into PCET square schemes.^63,64^ The wedge-like scheme shows the various pathways available to PCET while highlighting the formation and dissociation of the precursor and successor complexes. The new wedge-like scheme differs from that of Smith and co-workers in that every species in the four corners of a traditional PCET square scheme can undergo H-bond formation and dissociation, such that PT, ET, or even CPET could occur between H-bonded precursor and successor complexes.

Previous analyses of arylendiamine electrochemistry in the presence of weak H-bond acceptors were confronted with an apparent conundrum between the calculated Δ*G*_T_^°^ values and predicted H-bond strength. The analyses were based on the assumption that endergonic Δ*G*_T_^°^ implies the absence of PT proposed by Gupta and Linschitz for quinones.^32^ Based on this premise, a very strong H-bond between A^2+^-H_2_ and weak bases must form to account for the large *E*_1/2_^(2)^ shifts. However, this poses the question: how could a *very strong* H-bond form with a weak base? Support in favor of strong H-bond formation came at least in part from studies by Su and co-workers^61^ However, we note that the arylenediamines/imidazoles combinations could rather be described as of intermediate strength, borrowing from Linschitz’s classification. The conundrum dissipates in light of the proposed EEC_HB_C_PT_C_Diss_ mechanism. From our thermochemical analysis, previously overlooked endergonic successor complex dissociation reconciles exergonic H-bond formation and endergonic Δ*G*_T_^°^ (see Table 2 and Figure 2).

Moreover, our analysis shows that PT and H-bonding are not mutually exclusive; PT can occur even when Δ*G*_T_^°^ is low or even endergonic. A similar scenario likely operates in quinone electrochemistry as the driving forces for H-bonding and PT increase concurrently with Δp*K*_a_ between the H-bond and proton donor–acceptor pair. The biphasic dependence shown here arises from coupled equilibria and therefore can be modeled similarly to successive host–guest interactions, such as those between quinones and weak H-bond donors. While Uno and co-workers first proposed PT following formation of 1:2 adducts of quinones,^40^ deconvoluting H-bonding from PT and successor complex dissociation might be hindered by the formation of adducts with stoichiometries up to 1:6.^41–47^ Taken together, our studies suggest that standard nonaqueous PCET thermochemical analyses, which often rely on Nernstian relationships at one equivalent of acid or base,^74^ should be complemented by experiments designed to probe the continuum between H-bonding and PT. Potential shifts at one equivalent of acid or base could be due to equilibrium H-bonding or PT, or, even more likely, a point in their reactivity continuum. We recommend that neither H-bonding nor PT be excluded a priori when analyzing these systems.

Endergonic successor complex dissociation, acting as a thermodynamic and kinetic barrier, might be central to retaining chemical reversibility in the electrochemical waves as they shift with the added weak base. Chemical reversibility is retained upon addition of base despite initial deviations (for a complete discussion on the initial deviations, see SI). As shown, dissociation of the successor complex in the time scale of the experiment occurs only for the combination with the largest Δp*K*_a_ between A^2+^-H_2_ and base; but even then, it is only traceable when either the concentration of base is high or the scan rate is fast. The energy well formed by the C_HB_C_PT_C_Diss_ sequence maintains the equilibria of the electroactive species at the electrode, resulting in highly chemically reversible electrochemical waves. In addition to the large *E*_1/2_ shifts, the chemical reversibility of the waves is a central feature of quinone electrochemistry in aprotic media in the presence of weak H-bond donors. This reversibility in 2H^+^/2*e* ^–^ transfers is also a central feature of the biological function of quinones—a prime example is the Q-cycle,^75,76^ and it is also important in their use as redox mediators in electrocatalysis.^77^

## Conclusions

The studies of arylenediamine electro-oxidations in the presence of weak bases reported here show that the mechanism is EECCC, featuring proton transfer between the H-bonded precursor and successor complexes in solution. The role of successor complex dissociations, overlooked in previous studies, is found to be central for complete thermochemical and mechanistic analysis. Furthermore, the prevailing emphasis on H-bonding exclusively acting as a stabilizing effect at a low driving force is shown to be incomplete. H-bonding and proton transfer exist on a continuum; therefore, an “either/or” view between the H-bond and proton transfer implies an assumption that should be avoided. Finally, we propose that the energy well formed by the H-bonded precursor and successor complexes might explain the chemical reversibility displayed by arylenediamines and quinones. A deeper understanding of these effects could be essential for controlling charge transfer reversibility in synthetic and biological systems, for example, in redox-mediated electrocatalysis and the Q-cycle.

## Supplementary Material



## Notes

Synthesis, NMR spectra, electrochemical methods and analysis, DFT calculations and Hammett-type analysis (PDF)
