# Supplementary material for: Revisiting H‑Bond v. PT: The Role of Precursor and Successor Complexes in Intermolecular, Stepwise Proton-Coupled Electron Transfer
Source: ACS Omega. 2025 Oct 21;10(43):51396–405. doi: 10.1021/acsomega.5c06783 (PMC12593132; doi:10.1021/acsomega.5c06783)
Supplement: Supplementary file 1 [file ao5c06783_si_001.pdf]

Supporting information for:

Revisiting H-bond *v.* PT: The Role of Precursor and Successor Complexes in Inter-molecular, Stepwise PCET

Nikki Williams,[1] Tanay Parnaik,[1] Saptarshi Dutta,[1] Joseph Bergen,[1] Mauricio Cattaneo,[2] and Giovanny A. Parada.[1*]

[1] The College of New Jersey, Chemistry Department, Ewing, New Jersey 08628, USA

[2] INQUINOA (CONICET-UNT), Instituto de Química Física, Facultad de Bioquímica, Química y Farmacia, Universidad Nacional de Tucumán, Ayacucho 471 (4000), San Miguel de Tucumán, Argentina

[*]Correspondence to: paradag@tcnj.edu

Table of Contents.

1. Synthesis
2. NMR Spectra
3. Electrochemistry
4. DFT Calculations
5. Hammett-type analysis

1. Synthesis.

Materials: The following materials were used without any special purification: 2-(Dicyclohexylphosphino)-2',4',6'-tri-i-propyl-1,1'-biphenyl, XPhos, (Ambeed Inc), $Pd_2(dba)_3$ (Ambeed Inc), sodium tert-butoxide (Ambeed Inc), aniline (Ambeed Inc), 1,4-dibromobenzene (Ambeed Inc), 2,6-dibromonaphthalene (Ambeed Inc), Pd/C 10% w/w (Aldrich). N^4,$N^{4'}$-diphenyl-[1,1'-biphenyl]-4,4'-diamine, **3**–H_2, (Aldrich) was dried under a vacuum overnight with phosphorus pentoxide. All solvents for synthetic procedures were purified using a PureSolv Micro Multi Unit Solvent Purification System (Inert, Amesbury, MA) equipped with an Inert PureSolv Aluminum column Part #X730009 or purified by standard solvent purification methods.[1]

Instrumentation: Flash chromatography was done packing SINGLE StEP® Empty Columns (Thomson Instrument Company) with silica gel 230-400 mesh, Grade 6 (Fisher Scientific) run on a Biotage Isolera™ Flash Purification system. NMR was done on a Bruker (one Biospin Ascend and one Biospin Ultra Shield) 400 MHz spectrometer. LCMS was done on an Agilent 1260 Infinity LC-MS or Agilent 1260 Infinity II Quaternary System LC-MS.

N^1,N^4-diphenylbenzene-1,4-diamine (1–H_2): XPhos (27.3 mg, 0.057 mmol, 0.03 equiv), $Pd_2(dba)_3$ (35.2 mg, 0.038 mmol, 0.02 equiv), aniline (0.385 mL, 4.22 mmol, 2.2 equiv), 1,4-dibromobenzene (0.453 g, 1.92 mmol, 1 equiv), sodium *tert*-butoxide (0.406 g, 4.22 mmol, 2.2 equiv), and toluene (3.22 mL) were added to an oven-dried 2-5 mL Biotage® microwave vial equipped with a magnetic stir bar. The vial was capped with a rubber septum and bubbled under nitrogen at 160°C for 40 minutes. Upon termination of the reaction, the mixture was purified by automated silica gel column chromatography (n-hexane/ethyl acetate). Reductive workup with H_2 on Pd/C followed by solvent evaporation provided the product **1**–H_2. ^{1}H-NMR: (400 MHz, CD_2Cl_2) Figure S1: δ= 7.22 (dd, J = 8.5, 7.3 Hz, 4H, H_3, H_5, H_3', and H_5' diphenyl), 7.06 (s, 4H, H_2, H_3, H_5, and H_6 benzene), 6.98 (d, J = 8.6, 1.2 Hz, 4H, H_2, H_6, H_2', and H_6' diphenyl), 6.84 (ddd, J = 7.3, 1.2 Hz, 2H, H_4 and H_4' diphenyl), 5.67 (s, 2H, NH and NH'). ^{13}C-NMR: (400 MHz, CD_2Cl_2) Figure S2: δ= 145.00, 137.63, 129.67, 121.14, 120.25, 116.54 that correspond to 18 aromatic carbons given the symmetry of the molecule. MS (ESI): Calcd. for $C_{18}H_{16}N_2H^+$ $[M\text{-}H]^+$ 261.13, found 261.15 (100%).

N^2,N^6-diphenylnaphthalene-2,6-diamine (2–H_2): XPhos (23.1 mg, 0.048 mmol, 0.03 equiv), $Pd_2(dba)_3$ (29.5 mg, 0.032 mmol, 0.02 equiv), aniline (0.324 mL, 3.54 mmol, 2.2 equiv), 2,6-

dibromonaphthalene (0.4612 g, 1.61 mmol, 1 equiv), sodium *tert*-butoxide (0.3410 g, 3.54 mmol, 2.2 equiv), and toluene (3.22 mL) were added to an oven-dried 2-5 mL Biotage® microwave vial equipped with a magnetic stir bar. The vial was capped with a rubber septum and bubbled under nitrogen at 160°C for 40 minutes. Upon termination of the reaction, the mixture was purified by automated silica gel column chromatography (n-hexane/ethyl acetate). Reductive workup with H_2 on Pd/C followed by solvent evaporation provided the product **2**–H_2. ^{1}H-NMR: (400 MHz, CD_2Cl_2) Figure S3: δ= 7.60 (d, J = 8.7 Hz, 2H, H_4 and H_8 naphthalene), 7.42 (s, J = 2.3 Hz, 2H, H_1 and H_5 naphthalene), 7.28 (dd, J = 8.5, 7.2 Hz, 4H, H_3, H_5, H_3', and H_5' diphenyl), 7.21 (d, J = 8.7, 2.3 Hz, 2H, H_3 and H_7 naphthalene), 7.13 (d, J = 8.6, 1.2 Hz, 4H, H_2, H_6, H_2', and H_6' diphenyl), 6.93 (ddd, J = 7.2, 1.1 Hz, 2H, H_4 and H_4' diphenyl), 5.91 (s, 2H, NH and NH'). ^{13}C-NMR: (400 MHz, CD_2Cl_2) Figure S4: δ= 144.02, 139.42, 130.73, 129.75, 128.11, 121.47, 121.13, 117.80, 113.00 that correspond to 22 aromatic carbons given the symmetry of the molecule. Calcd. for $C_{22}H_{18}N_2H^+$ $[M\text{-}H]^+$ 311.15, found 311.18 (100%).

N^4,$N^{4'}$-diphenyl-[1,1'-biphenyl]-4,4'-diamine (3–H_2**):** ^{1}H-NMR: (400 MHz, CD_2Cl_2) Figure S5: δ= 7.50 (d, J = 8.6 Hz, 4H, H_2, H_6, H_2', and H_6' biphenyl), 7.28 (dd, J = 8.5, 7.3 Hz, 4H, H_3, H_5, H_3', and H_5' diphenyl), 7.14 (d, J = 8.7 Hz, 4H, H_3, H_5, H_3', and H_5' biphenyl), 7.11 (d, J = 8.6, 1.1 Hz, 4H, H_2, H_6, H_2', and H_6' diphenyl), 6.93 (ddd, J = 7.4, 1.2 Hz, 2H, H_4 and H_4' diphenyl), 5.87 (s, 2H, NH and NH'). ^{13}C-NMR: (400 MHz, CD_2Cl_2) Figure S6: 1δ= 143.57, 142.40, 133.79, 129.73, 127.58, 121.30, 118.34, 118.05 that correspond to 24 aromatic carbons given the symmetry of the molecule. Calcd. for $C_{24}H_{20}N_2H^+$ $[M\text{-}H]^+$ 337.16, found 337.19 (100%).

2. NMR Spectra.

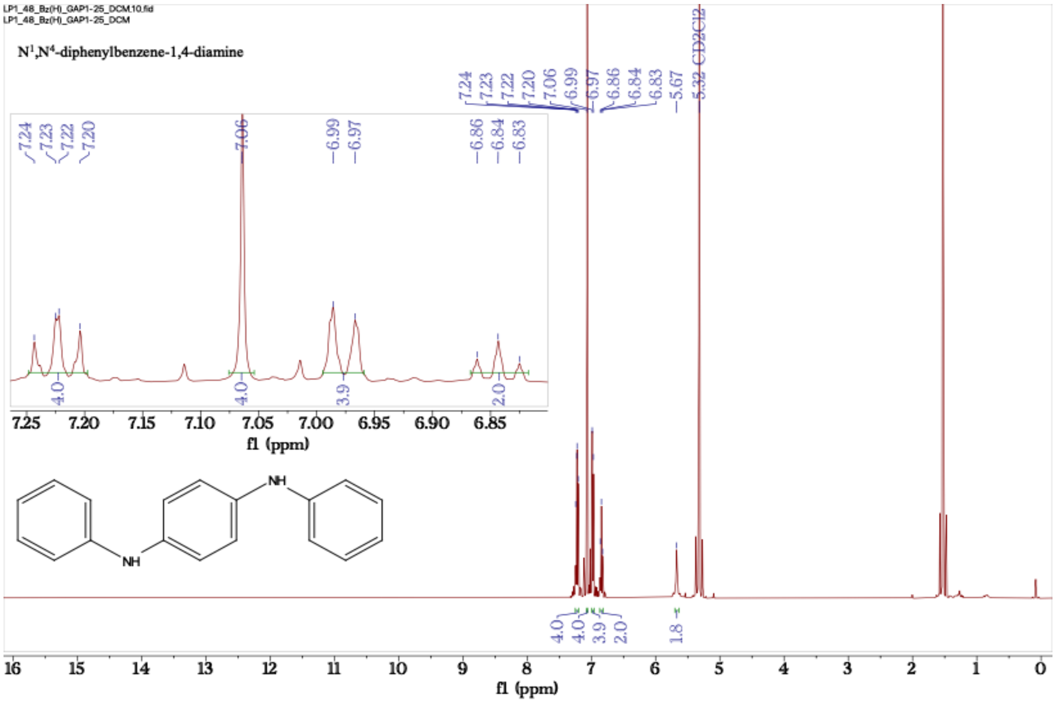

Figure S1. ^{1}H NMR spectrum of N^1,N^4-diphenylbenzene-1,4-diamine (**1**–H_2).

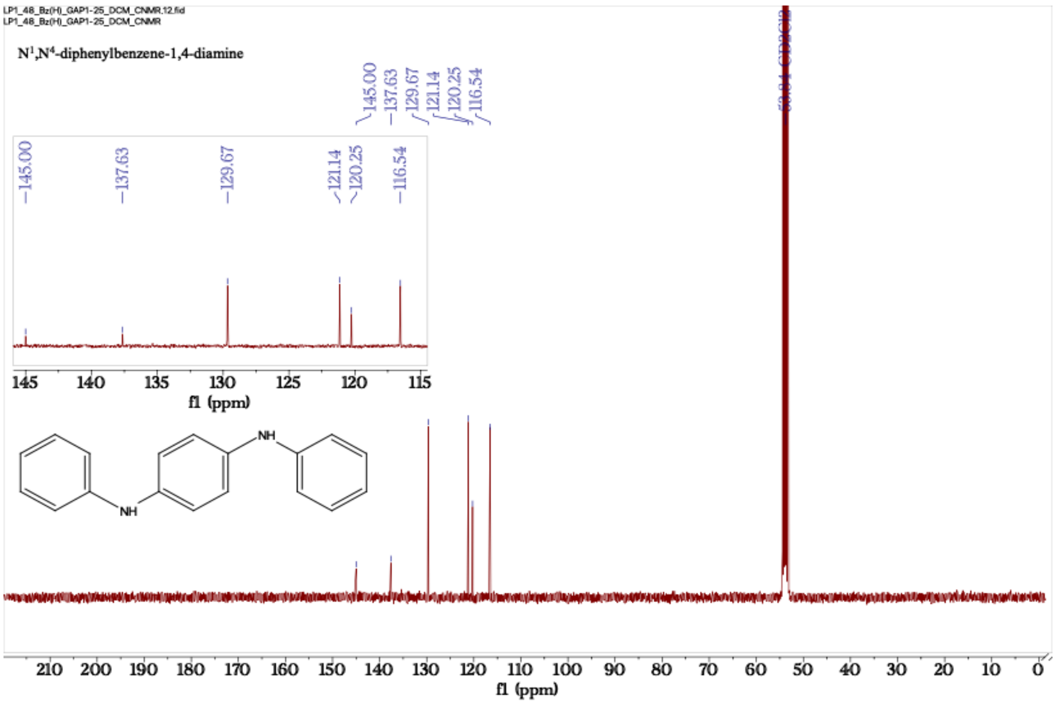

Figure S2. ^{13}C NMR spectrum of N^1,N^4-diphenylbenzene-1,4-diamine (**1**–H_2).

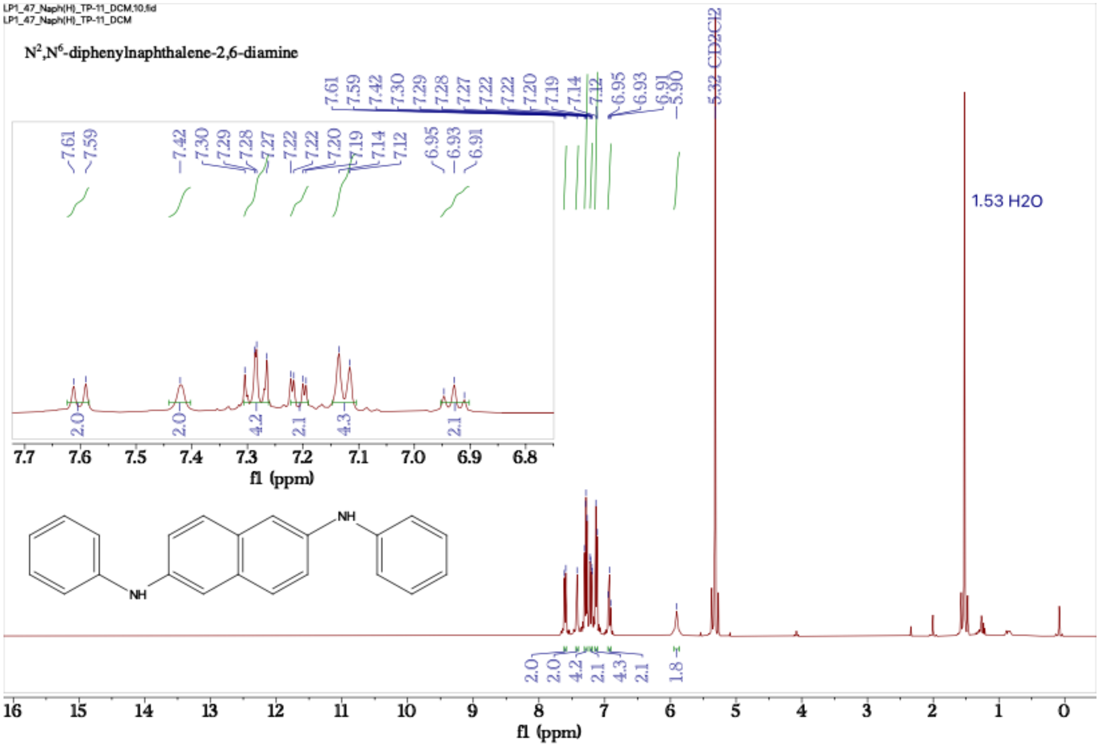

Figure S3. ^{1}H NMR spectrum of N^2,N^6-diphenylnaphthalene-2,6-diamine (**2**–H_2).

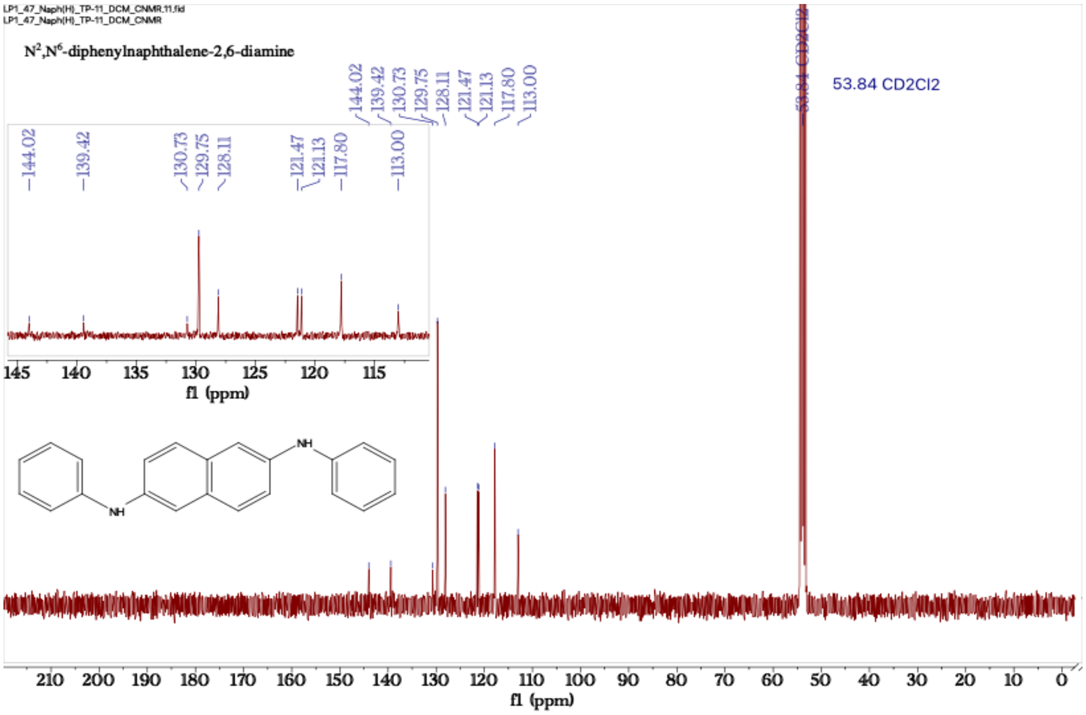

Figure S4. ^{13}C NMR spectrum of N^2,N^6-diphenylnaphthalene-2,6-diamine (**2**–H_2).

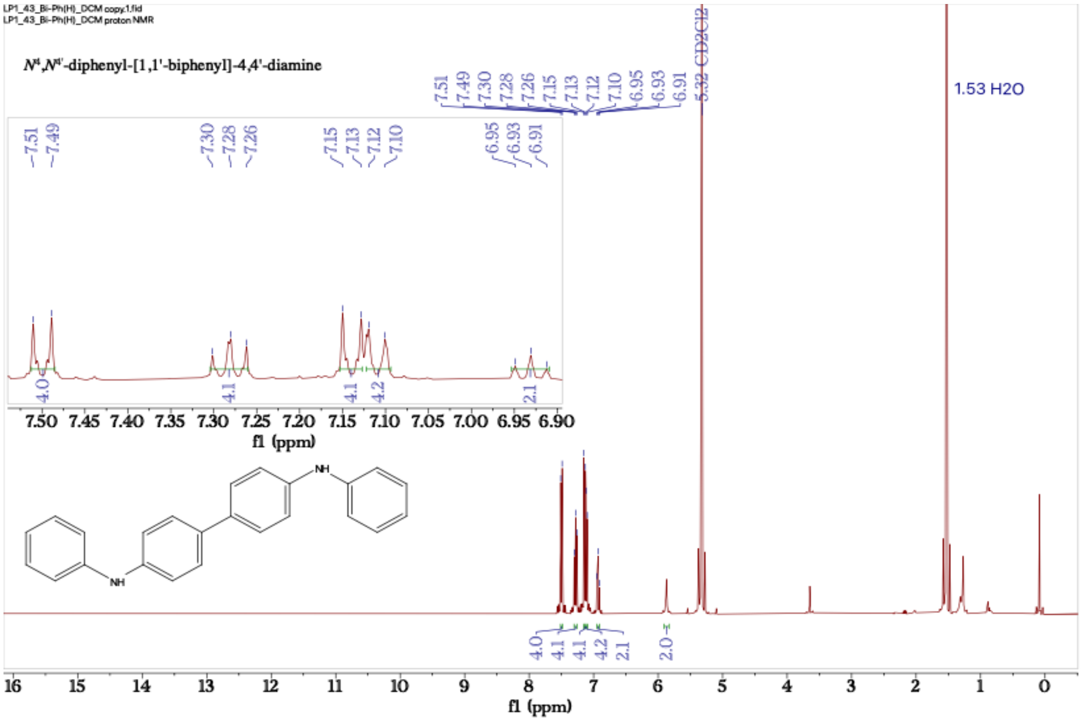

Figure S5. ^{1}H NMR spectrum of $N^4,N^{4'}$-diphenyl-[1,1'-biphenyl]-4,4'- diamine (**3**–H_2).

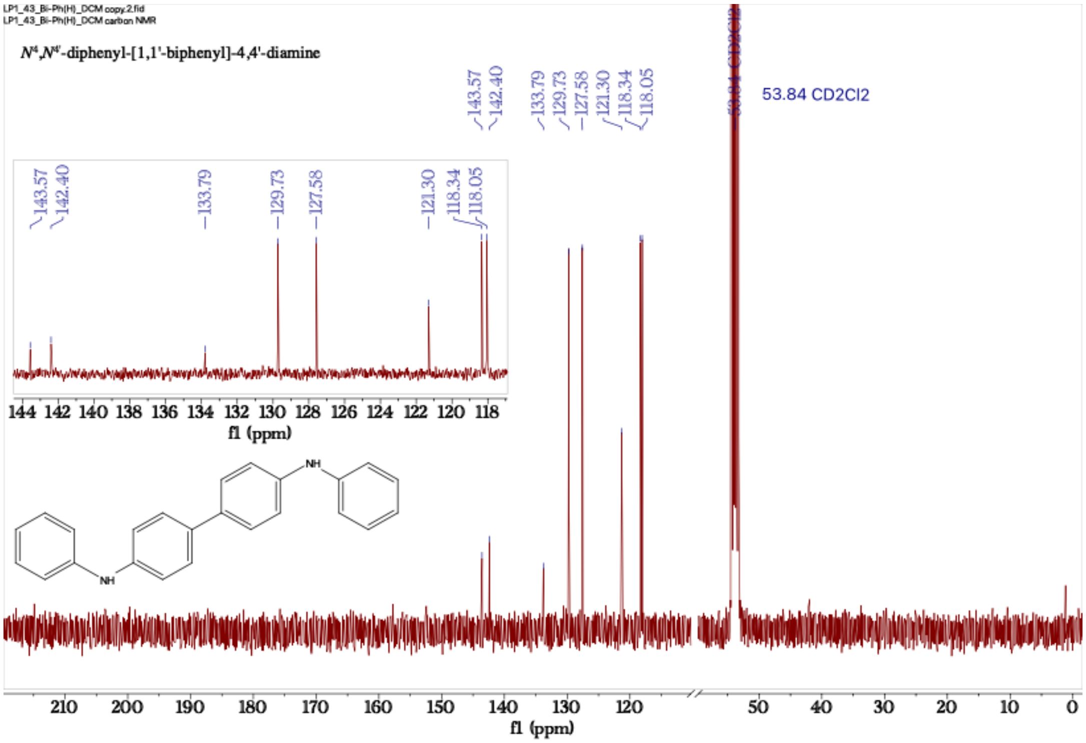

Figure S6. ^{13}C NMR spectrum of $N^4,N^{4'}$-diphenyl-[1,1'-biphenyl]-4,4'- diamine (**3**–H_2).

3. Electrochemistry

Materials: The electrolyte, NBu_4PF_6 (Ambeed Inc), was recrystallized from 95% ethanol by heating a mixture of 25 g NBu_4PF_6 in 100 mL of ethanol in a 250-mL round bottle flask until a homogeneous solution is attained. The flask was seal with a septum, cool off to room temperature and placed at –20°C overnight. The electrolyte was then filtered on a frit and washed with a small amount of ethanol. The procedure was repeated three times. After that, the electrolyte was vacuum-dried in a round bottom flask placed on the Schlenk line at no less than 100 mTorr and on an oil bath at 100 °C overnight. HPLC-grade CH_3CN (Aldrich) was filtered through an activated alumina column (Inert PureSolv Aluminum column, #X730009) on a PureSolv Micro Multi Unit Solvent Purification System (Inert, Amesbury, MA) and collected under a N_2 atmosphere. High-purity molecular sieves (Fluka product no. 69832), activated by heating at 250 °C, were added to the CH_3CN two days prior to use. Arylenediamines **1**-H_2, **2**-H_2 and **3**-H_2 and bases 3,5-dichloropyridine (Aldrich), pyrazine (Aldrich) and 4-cyanopyridine (Aldrich) were vacuum-dried on the Schlenk line at no less than 100 mTorr for 48 hours in the presence of phosphorus pentoxide (Aldrich), and store in a desiccator under dynamic vacuum and phosphorus pentoxide. Ferrocene (Ambeed Inc) was sublimated and used to reference the potential. N_2 99.999% purity (AGA) was used in all procedures.

Instrumentation: Electrochemical experiments were done using a WaveDriver 10 or Wavedriver 20 Potentiostats/Galvanostats (Pine Instruments, Duran, NC). Three electrodes electrochemical cells with Teflon tops and working (standard 3.0 mm diameter GC), pseudo-reference (Ag wire inside a glass tube, filled with electrolyte solution, with a porous vycor tip) and counter electrodes (Pt) were used (Bioanalytical Systems, Inc., West Lafayette, IN). Working electrodes were polished in aqueous suspensions of 1.0, 0.3, and 0.05 μm (Allied High Technology Products, Inc. Rancho Dominguez, CA) on Alumina polishing pads (Bioanalytical Systems, Inc., West Lafayette, IN). UV-vis spectra were performed using an Agilent Cary 100 spectrophotometer. Arylenediamines and bases were weighted using a Mettler Toledo UMX2 ultra-microbalance. The humidity in the laboratory was minimized with a Dri-Eaz Revolution LGR Dehumidifier.

Voltametric Titration Procedure: CH_3CN in a bubbler equipped with glass frit and loaded with activated molecular sieves was sparged with N_2 for 20 mins. The electrochemical cell was set up with an oven dried cell and freshy polished, cleaned electrodes. 3.00 mL of electrolyte NBu_4PF_6

0.1 M in CH_3CN were added to the cell. The cell was sealed with parafilm and the electrolyte was sparged with N_2 for 10 mins. N_2 flow was maintained in the headspace of the cell if N_2 was not sparing the electrolyte. Cyclic voltammograms (CV) in a wide potential range (typically from – 1.5 V to 1.0 V vs Fc) were collected at 200 mV/s (our freshly prepared pseudo-reference electrodes are typically ~ 200 to 300 mV vs Fc). If no Faradaic currents are observed, 20 scans were collected; otherwise, fresh electrolyte and cell were prepared. IR drop was measured (typically from 80 to 130 Ω) for compensation using the positive feedback method. A 0.50 mM solution of the arylenediamines was prepared in the cell to a final volume of 5.00 mL by addition of a stock solution of the arylenediamines prepared in electrolyte. The cell was sparged with N_2 for 10 mins. A potential window for the experiments was established by CV. A 100.0 μL aliquot of the cell solution was taken to prepare a solution to verify the final concentrations of arylenediamines in the cell by UV-Vis using pre-determined calibration curves. Using the concentration of arylenediamines in the cell, solutions of the base were prepared such that 20.0 μL of the solutions deliver 0.20, 1.00, and 20.0 equivalents, with respect to the total arylenediamine in the cell. These solutions covering different concentrations are preferred to avoid large volume changes in the cell. Nonetheless, volume corrections in the data workup are always required to calculate accurate concentrations. CVs were recorded at various scan rates from 100 to 12000 mV/s for each base addition from 0.20 to 200 equivalents with respect to the total arylenediamine in the cell. A new cell with a freshly polished working electrode and thoroughly CH_3CN rinsed and dried pseudo-reference and counter electrode with 3.00 mL of electrolyte was used to record backgrounds at all scan rates in the potential window of the experiment. A small crystal of Fc was added to the electrolyte and a CV at 200 mV/s was recorded. Each experiment was at least run on triplicates. The difference between the potentials for the first and second oxidation of the arylenediamines ($\Delta E_{1/2} = E_{1/2}^{(1)} - E_{1/2}^{(2)}$) in absence of base was monitored between replicate experiments. Lower than expected $\Delta E_{1/2}$ values were taken as a sign of moisture contaminations in the cell. **1**–H_2 was the only compound that showed a significant sensitivity to moisture contamination. Addition of activated molecular sieves in the cell typically was enough to correct small moisture contaminations.

Data workup: Python scripts were written to process the data minimizing human error. The voltammograms were Fc corrected and background subtracted prior to peak peeking based on first derivatives and data interpolation to zero to find current maxima.

Data analysis showed that only the first additions in the 2 - 200 equivalents range could be used in the plots of $\exp(f\Delta E_{1/2}) - 1$ vs. [Base] and $\log_{10}(\exp(f\Delta E_{1/2}) - 1)$ vs. $\log_{10}$([Base]). This is because when $E_{1/2}^{(2)}$ shifts close to $E_{1/2}^{(1)}$ the overlapping waves cause unreliable determination of the peak potentials. The effect is more significant when K_{obs} is large and the $\Delta E = E_2^\circ - E_1^\circ$ (in absence of base) is small.

UV-Vis-NIR spectra of the arylenediamines and their mono-cations.

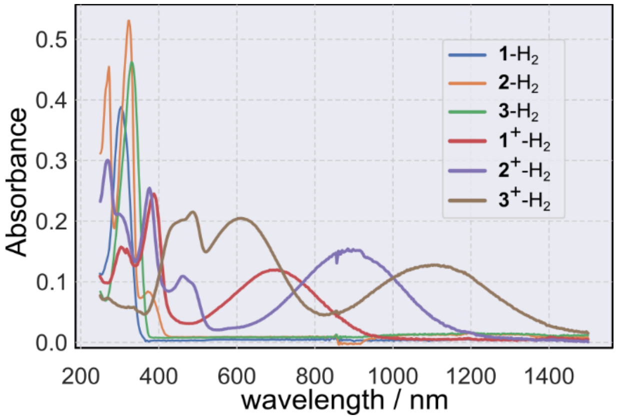

Figure S7. UV-Vis-NIR spectra of the arylenediamines and their mono-cations. The latter are produced by electrolysis. The electrolysis potential is set 50 mV positive of $E_{1/2(1)}$ unit, until the spectra stop changing. The spectra of the mono-cations show the expected IV-CT bands. Their analysis using ET and PCET theories is part of a study beyond the scope of the present one.

Deviations from chemical reversibility: Similarly to quinones,[2] CVs of arylenediamines in aprotic media show deviations of chemically reversibility in the second oxidation with $1 > i_a/i_c$ and smaller currents than those observed for the first oxidation. The effect has been documented in several studies, for example by Giordano *et al.*,[3] Compton *et al.*[4] and Smith *et al.*[5] each with different mechanistic proposals. Smith *et al.* proposed an adduct, a hetero-dimmer, held by H-bonding and π-π stacking form after the first oxidation. The proposed adduct is stable and does not undergo heterogeneous ET under the potential window of the experiment. However, it dissociates under millimolar concentrations of A–H_2 to form the dications A^{2+}–H_2. The arylenediamines studied show similar deviations of chemical reversibility, particularly pronounced for **1**–H_2. The adduct's stability has been shown to depend on the electrode material. For **1**–H_2, the deviations to reversibility are more pronounced in Pt over GC (Figure S8), and therefore we preferred the latter in our studies.

We assume that the deviations from chemical reversibility do not affect our treatment of coupled equilibria. This is because, as shown in this study, 1) weak bases interact first in H-bonding with the dications A^{2+}–H_2, not the mono-cation $A^{\bullet+}$–H_2, 2) according to Smith *et al.*[5] the dications A^{2+}–H_2 is indeed the product of the second oxidation and we have not evidenced in our studies to believe otherwise, and 3) experimental equilibrium constants from titrimetric studies result from the extent of $E_{1/2}^{(2)}$ shifts per base concentration with respect to potentials in absence of base; that is, from potential differences, not absolute potentials.

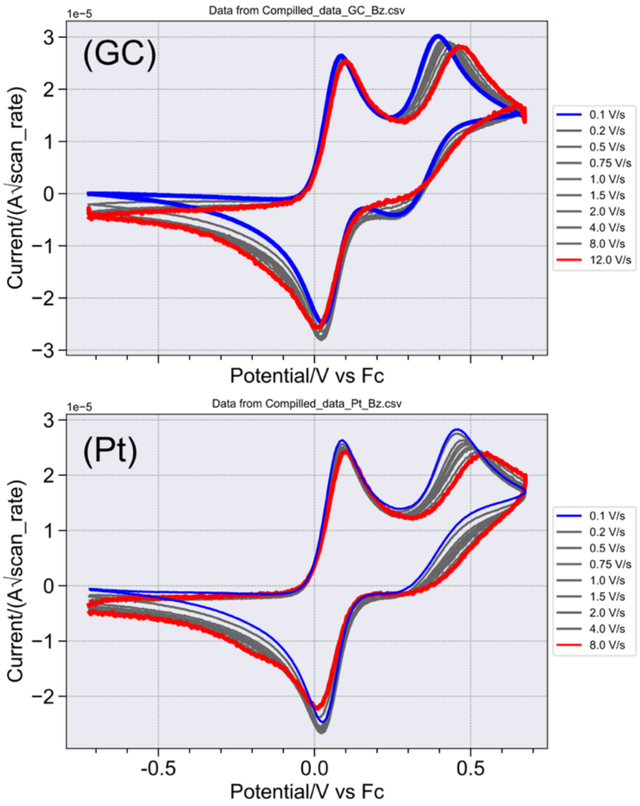

Figure S8. Background subtracted **1**–H_2 CVs at different scan rates (ν), with currents normalized by $\nu^{1/2}$, in glassy carbon (GC) and platinum electrode (Pt).

Unlike **1**–H_2 and **3**–H_2, **2**–H_2 shows additional features besides $E_{1/2}^{(1)}$ and shifting $E_{1/2}^{(2)}$ in the presence of weak bases. The appearance of the new features depends on the strength of the base, the stronger the base the lower the number of equivalents it takes for the features to appear. An additional oxidation wave appears between $E_{1/2}^{(1)}$ and $E_{1/2}^{(2)}$, at around 0.3 V vs Fc, as well as a reduction at significantly more cathodic potentials –0.6 V vs Fc. The latter shifts more catholically the stronger the base. The new reductive wave grows at the expense of the reduction associated with $E_{1/2}^{(1)}$. With

the weaker 3,5-diCl-py, these are the only additional features up to 300 equivalents, Fig S13 panel A, but additional features appear with stronger bases pyrz and 4CN-py, Fig S13 panels B and C.

Although we do not have direct evidence to explain the behavior, we speculate that the reactivity is due to differences in charge localization in the naphtalen aminyl radical compared to the benzene and diphenyl analogs. The deprotonated aminyl radical (H–$N^{\bullet+}$–napth–$N^{\bullet}$, using the nomenclature of Scheme 2 panel B in the main text), formed after double oxidation and deprotonation might display unique (enhanced) reactivity compared to the benzene and diphenyl analogs,[6] such as inter or intra HAT abstraction or radical substitutions.[7]

$E_{1/2}^{(2)}$ shifts before and after 2 equivalents of base per 1–H_2.

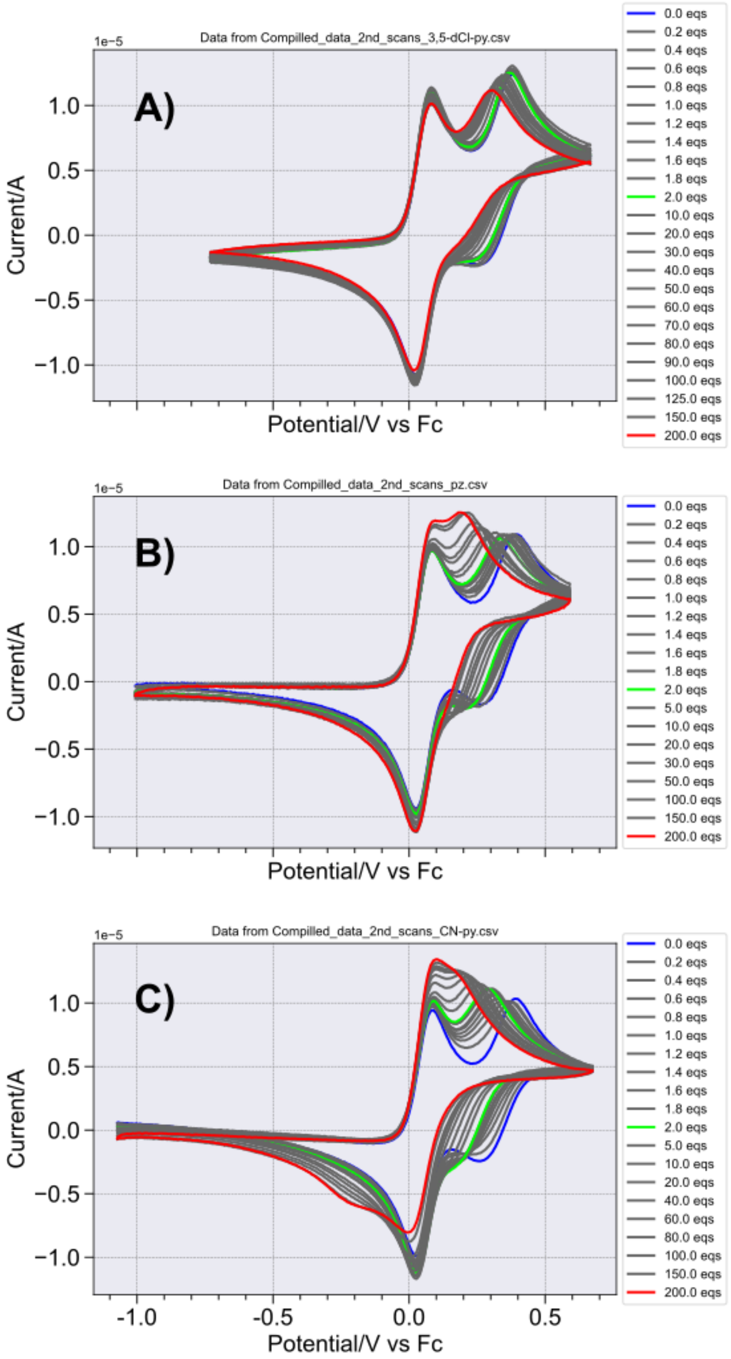

Figure S9. Background-subtracted CVs for **1**-H_2 (0.5 mM) in 0.1 M NBu_4PF_6 in CH_3CN with and without added (A) 3,5-diCl-py, (B) pyrz and (C) 4CN-py at 200 mV/s. Without added base (in blue) and with successive added base from sub-stoichiometric to a large excess up to 200 equivalents (in red).

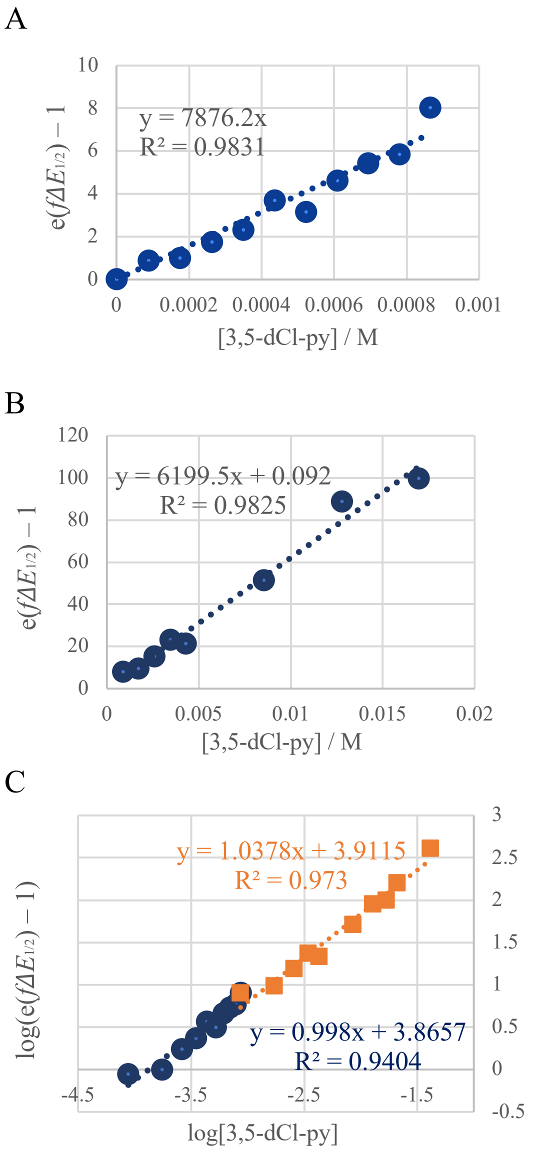

Figure S10. Change in $E_{1/2}$ for the second oxidation of **1**–H_2 with increasing amounts of 3,5-dCl-py, A) before 2 equivalents of added base, B) after 2 equivalents of added base, C) per decade change before (blue) and after (orange) 2 equivalents.

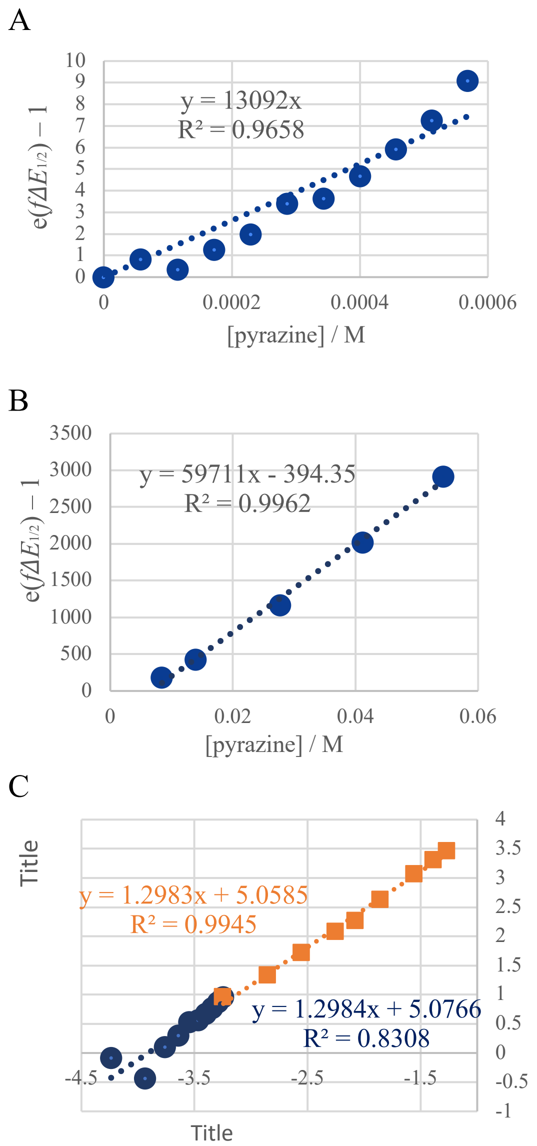

Figure S11. Change in $E_{1/2}$ for the second oxidation of **1**–H_2 with increasing amounts of pyrazine, A) before 2 equivalents of added base, B) after 2 equivalents of added base, C) per decade change before (blue) and after (orange) 2 equivalents.

A

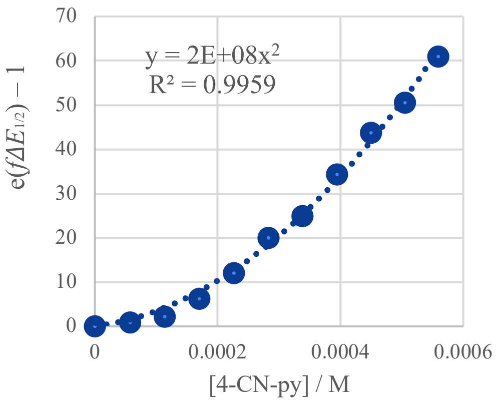

B

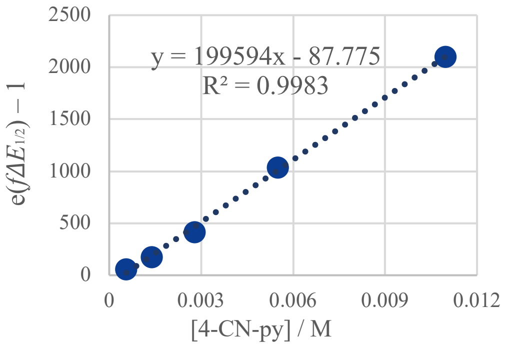

C

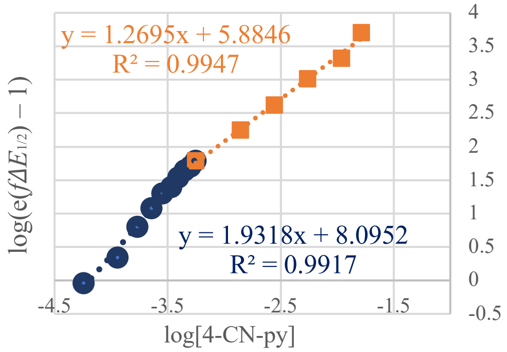

Figure S12. Change in $E_{1/2}$ for the second oxidation of **1**–H_2 with increasing amounts of 4-CN-py, A) before 2 equivalents of added base, B) after 2 equivalents of added base, C) per decade change before (blue) and after (orange) 2 equivalents.

$E_{1/2}^{(2)}$ shifts before and after 2 equivalents of base per 2–H_2.

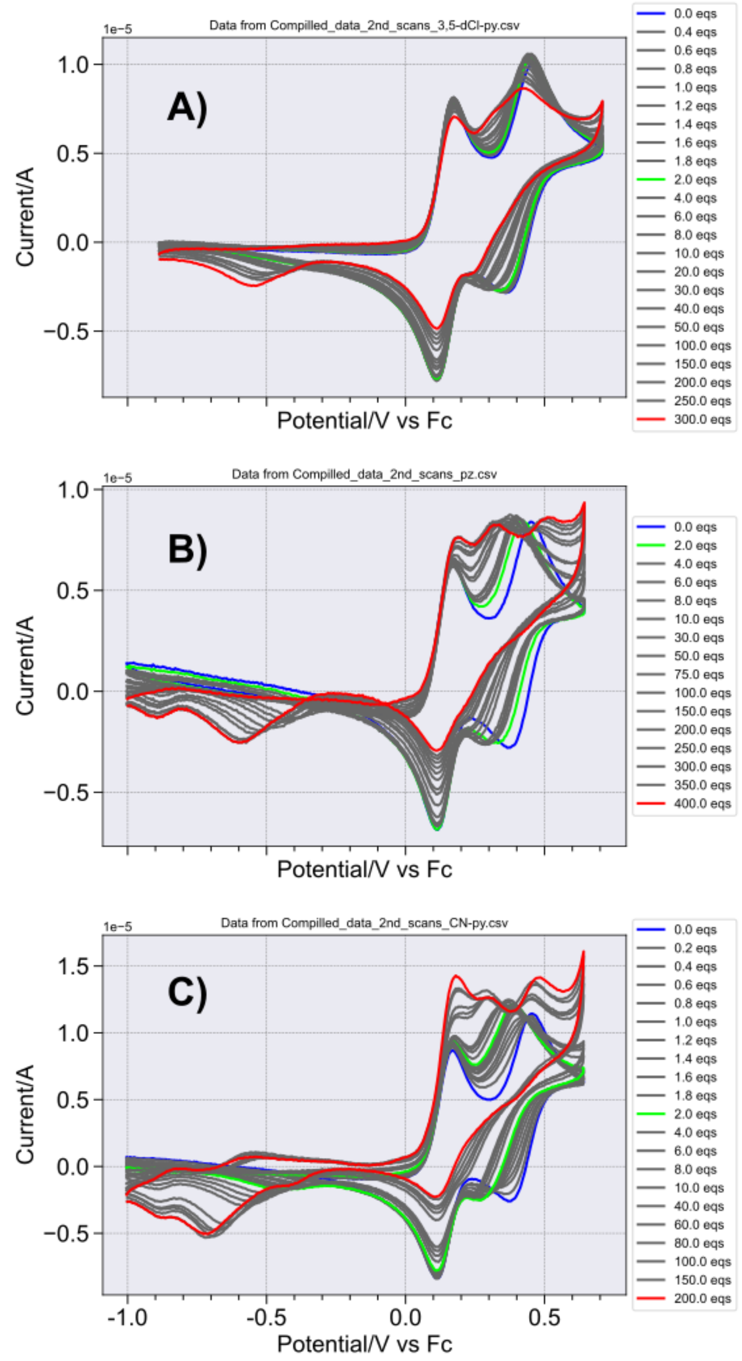

Figure S13. Background-subtracted CVs for **2**-H_2 (0.5 mM) in 0.1 M Nbu_4PF_6 in CH_3CN with and without added (A) 3,5-diCl-py, (B) pyrz and (C) 4CN-py at 200 mV/s. Without added base (in blue) and with successively added base from sub-stoichiometric to a large excess up to 400 equivalents (in red). See the section "Deviations from chemical reversibility" above for a discussion of the new features that arise as the strength and concentration of base increase.

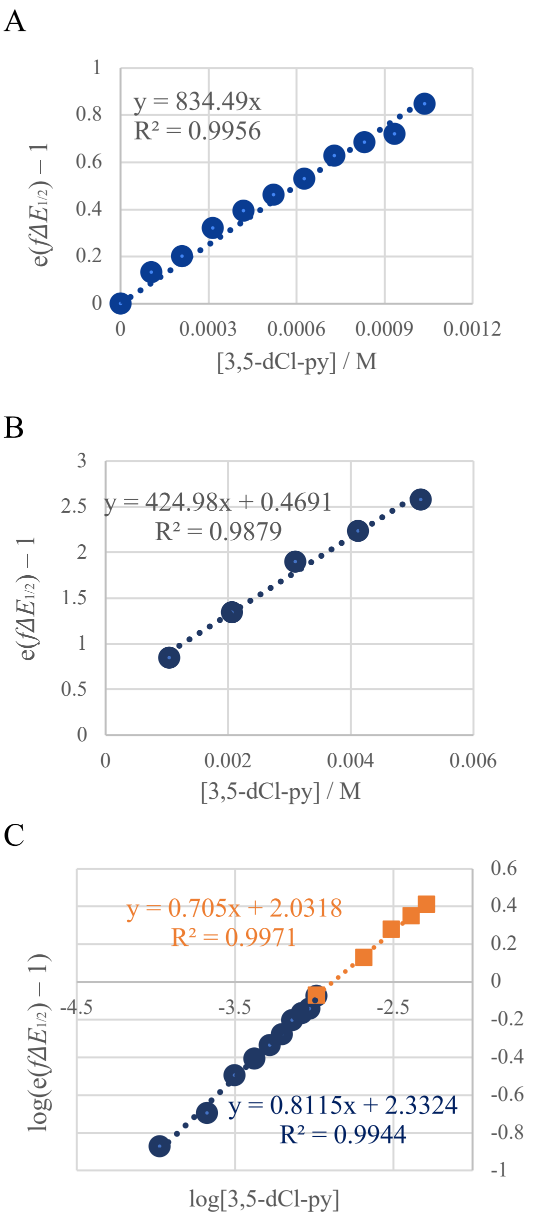

Figure S14. Change in $E_{1/2}$ for the second oxidation of **2**–H_2 with increasing amounts of 3,5-dCl-py, A) before 2 equivalents of added base, B) after 2 equivalents of added base, C) per decade change before (blue) and after (orange) 2 equivalents.

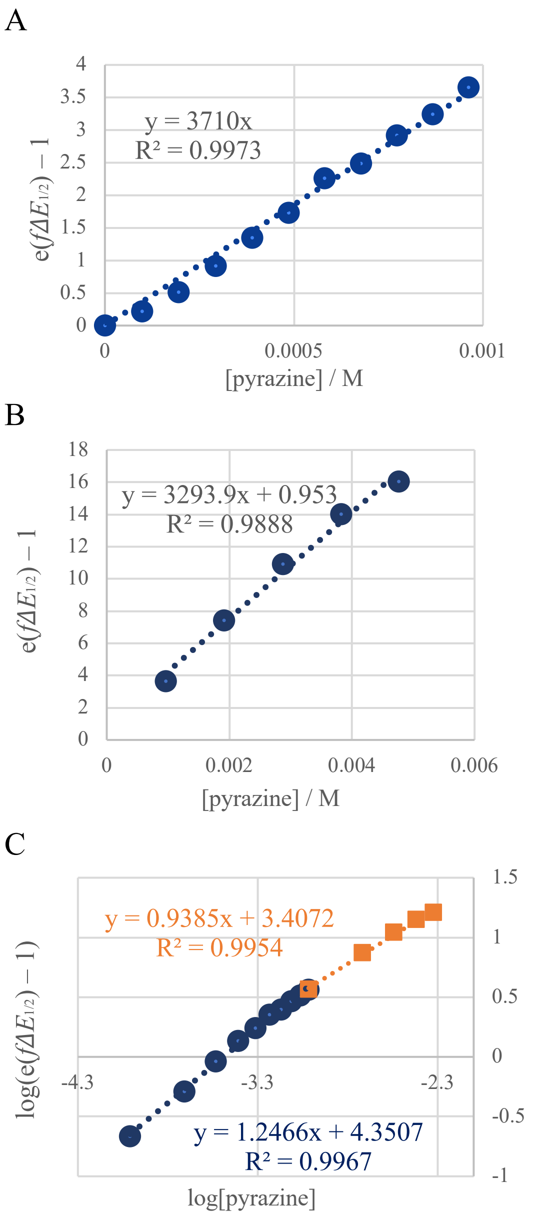

Figure S15. Change in $E_{1/2}$ for the second oxidation of **2**–H_2 with increasing amounts of pyrazine, A) before 2 equivalents of added base, B) after 2 equivalents of added base, C) per decade change before (blue) and after (orange) 2 equivalents.

A

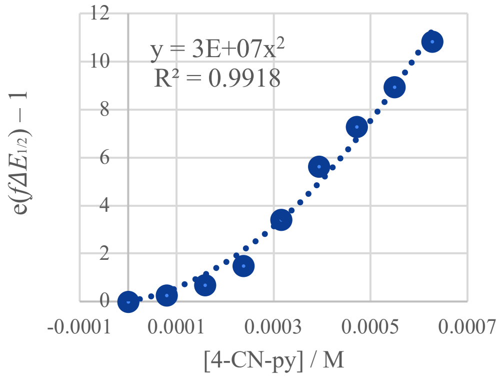

B

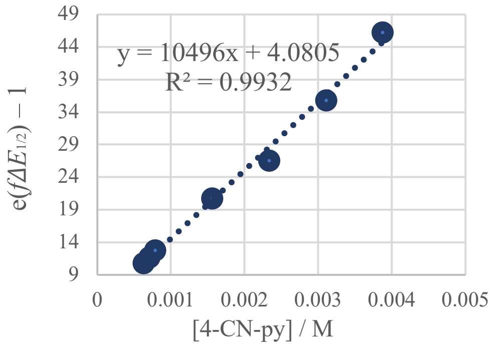

C

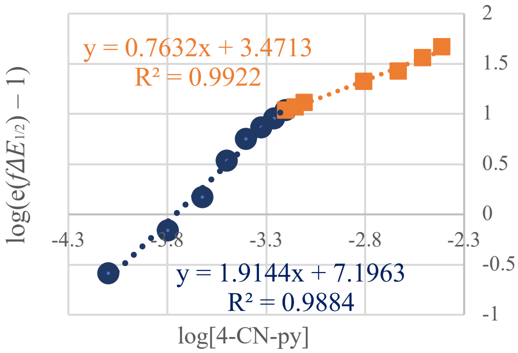

Figure S16. Change in $E_{1/2}$ for the second oxidation of **2**–H_2 with increasing amounts of 4-CN-py, A) before 2 equivalents of added base, B) after 2 equivalents of added base, C) per decade change before (blue) and after (orange) 2 equivalents.

$E^{(2)}_{1/2}$ shifts before and after 2 equivalents of base per 3–H_2.

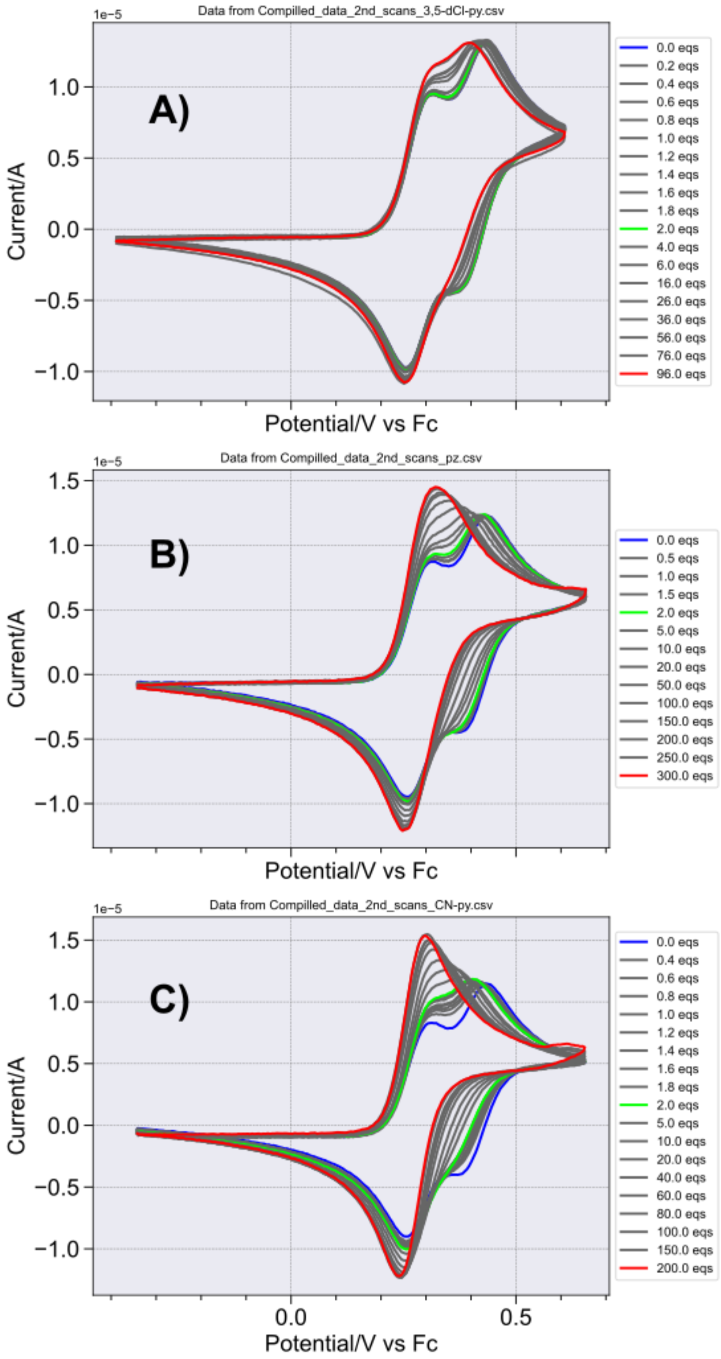

Figure S17. Background-subtracted CVs for **3**-H_2 (0.3 mM) in 0.1 M NBu_4PF_6 in CH_3CN with and without added (A) 3,5-diCl-py, (B) pyrz and (C) 4CN-py at 200 mV/s. Without added base (in blue) and with successively added base from sub-stoichiometric to a large excess up to 300 equivalents (in red).

The merging of the two oxidation waves for **3**-H_2 in the presence of pyrz and 4-CN-py (Figure 1H and 1I) is a direct visual consequence of the large cathodic shift of $E_{1/2(2)}$. The combination of 3-

H_2, the arylenediamine with the smallest $\Delta E_{1/2}$, and the strongest bases shows shifts that are large enough such that the two waves overlap and appear as a single 2$e-$ wave. This behavior is fully consistent with our model and represents the limiting case of a very large potential shift driven by the coupled chemical reactions. The "Data workup" section above describes the criteria to perform the quantitative analysis of the $E_{1/2(2)}$ shifts accounting for this effect.

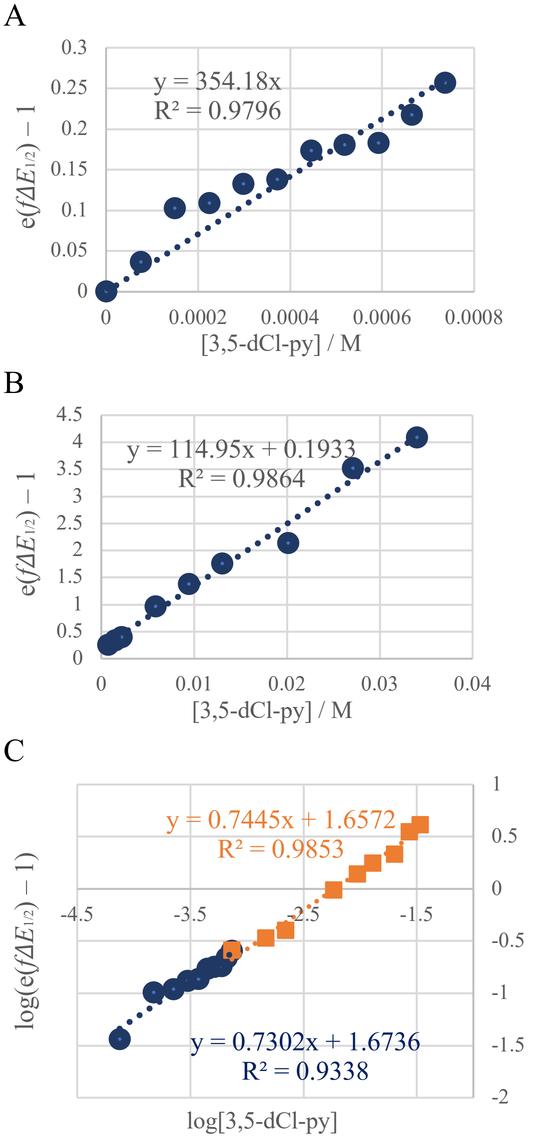

Figure S18. Change in $E_{1/2}$ for the second oxidation of **3**–H_2 with increasing amounts of 3.5-dCl-py, A) before 2 equivalents of added base, B) after 2 equivalents of added base, C) per decade change before (blue) and after (orange) 2 equivalents.

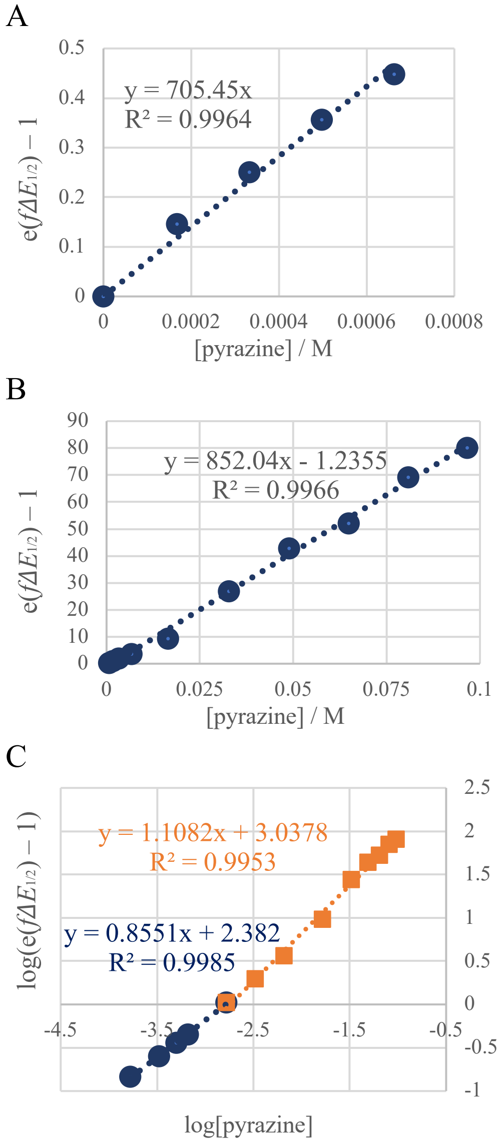

Figure S19. Change in $E_{1/2}$ for the second oxidation of **3**–H_2 with increasing amounts of pyrazine, A) before 2 equivalents of added base, B) after 2 equivalents of added base, C) per decade change before (blue) and after (orange) 2 equivalents.

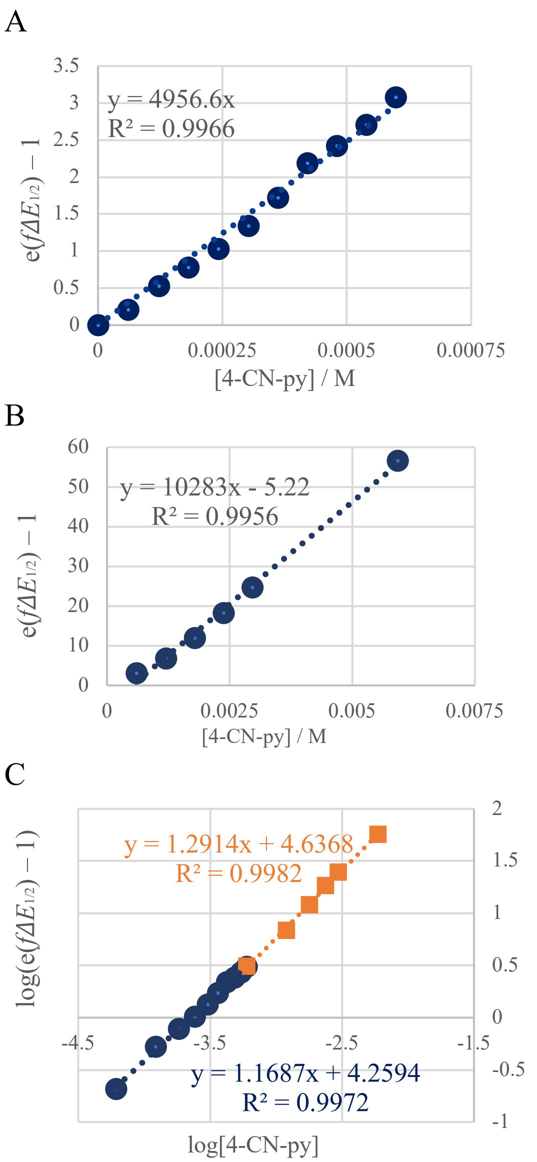

Figure S20. Change in $E_{1/2}$ for the second oxidation of **3**–H_2 with increasing amounts of 4-CN-py, A) before 2 equivalents of added base, B) after 2 equivalents of added base, C) per decade change before (blue) and after (orange) 2 equivalents.

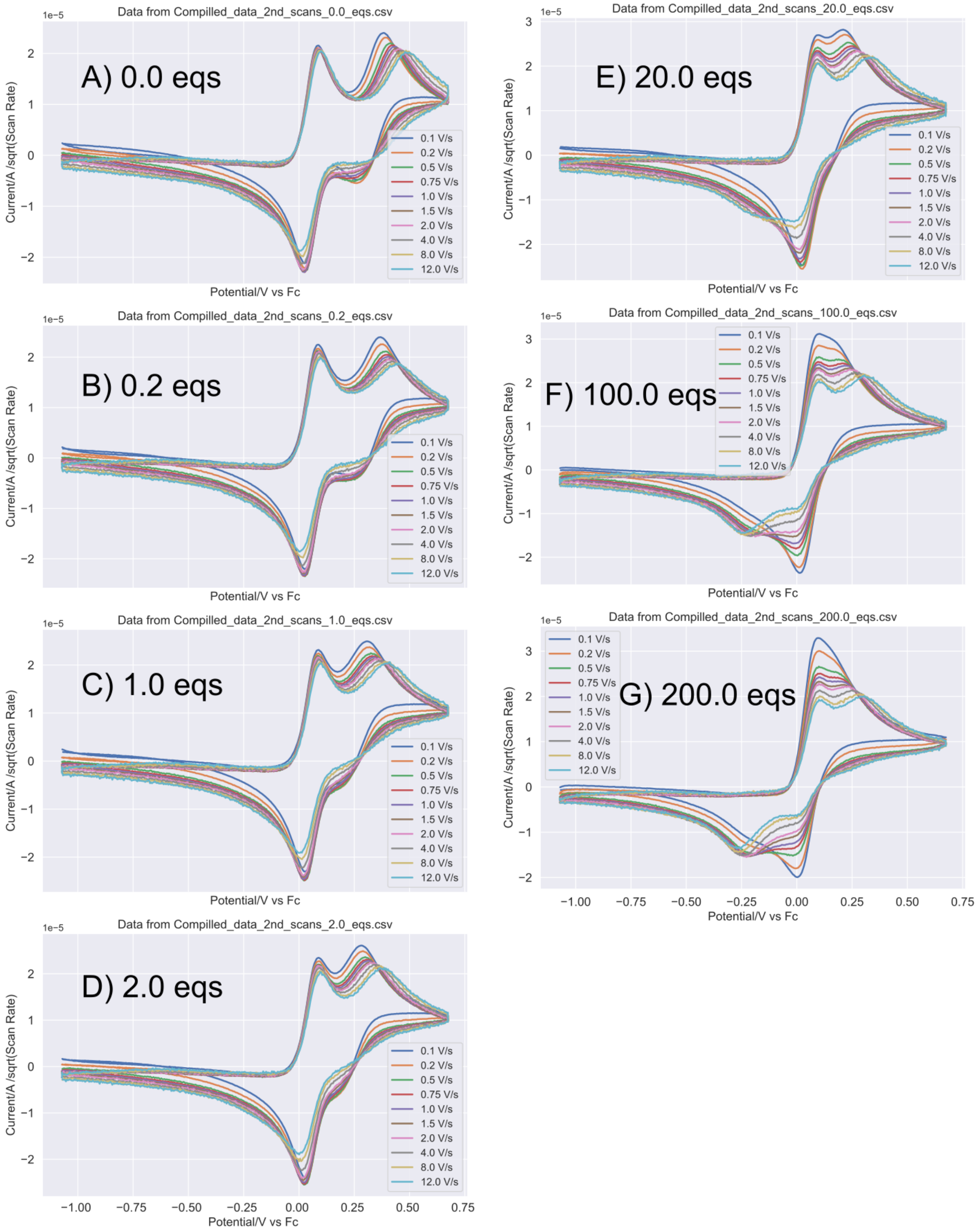

Figure S21. Background subtracted CVs of **1**–H_2 (0.5 mM) in 0.1 M NBu_4PF_6 in CH_3CN with increasing amounts of 4-CN-py at different scan rates (ν), with currents normalized by $\nu^{1/2}$. The number of equivalents of 4-CN-py is varied as follows: A) 0.0 equivalents (eqs), B) 0.2 eqs, C) 2.0 eqs, D) 20.0 eqs, E) 100.0 eqs, F) 200.0 eqs.

4. DFT Computations.

Calculations were carried out using Gaussian 09 software package.[8] Geometry optimization of **1**-H_2, **2**-H_2 and **3**-H_2 and their mono cations and dications were performed using a standard methodology[9] with density functional theory (DFT). To benchmark redox potentials and pK_a's several molecules were used to include corrections on absolute calculated values. For acidities, the bases used were: pyrazine, 4-CN-pyridine, pyridine, 2,4,6-Me_3-pyridine, triethyl-amine, DBU, and TBD.[10] For arylenediamines, the values for **1**–H_2, $\mathbf{1}^{\bullet+}$–H_2 and $\mathbf{1}^{2+}$–H_2 in DMSO[11] were used to calculate the corresponding values in MeCN using thermochemical cycles, [12] and used as one point benchmark to correct the values for **2**–H_2, $\mathbf{2}^{\bullet+}$–H_2, $\mathbf{2}^{2+}$–H_2, **3**–H_2, $\mathbf{3}^{\bullet+}$–H_2 and $\mathbf{3}^{2+}$–H_2. All molecules were calculated with a B3LYP[13] functional with 6-311++G(d,p)[14] basis set including diffuse functions, following similar methodology reported for quinones.[9b] Depending on the spin multiplicity restricted or unrestricted formalism was considered. Solvent effects were computed by the SMD[15] model in MeCN. Other functionals, dispersion correction and basis sets were considered to adjust methodology, including hybrid, range-separated hybrid and double-hybrid functional: BP86/aug-cc-pVQZ, B3LYP-D3/def2tzvpp, CAM-B3LYP/6-311++G(d,p), M06-2x/def2tzvpp, ωB97xd/def2tzvpp, and PW6B95D3/def2tzvpp.[16] Figure S22 summarizes the result of the different methods for **1**–H_2, $\mathbf{1}^{\bullet+}$–H_2 and $\mathbf{1}^{2+}$–H_2, with assigned p$K_{a(1)}$, p$K_{a(2)}$ and p$K_{a(3)}$. Vibrational frequencies were computed to verify whether every optimized geometrical structure is an energy minimum, and to evaluate its zero-point vibration energy (ZPVE). Thermochemical data were calculated at T = 298.15 K, and all calculated free energies included zero-point energy, entropic contributions, and solvation effects. Calculated absolute pK_a values were obtained following: pK_a = $\Delta G_{(solv)}/2.303RT$, where $\Delta G_{(solv)}$ is the free energy difference between the protonated and deprotonated form.[17] The values of G_{gas} (H^+) = -6.28 kcal/mol, and ΔG_{solv} (H^+) = 255.1 kcal/mol were considered for pK_a calculations.[18] The relative pK_a values were calculated relative to the corresponding values for the average correction obtained with the reference species using isodesmic reactions. This procedure is equivalent to determining the difference between the experimental and calculated values for the reference species and adding this correction to all calculated values. The resulting pK_a values are quantitatively accurate for structurally similar species.[17]

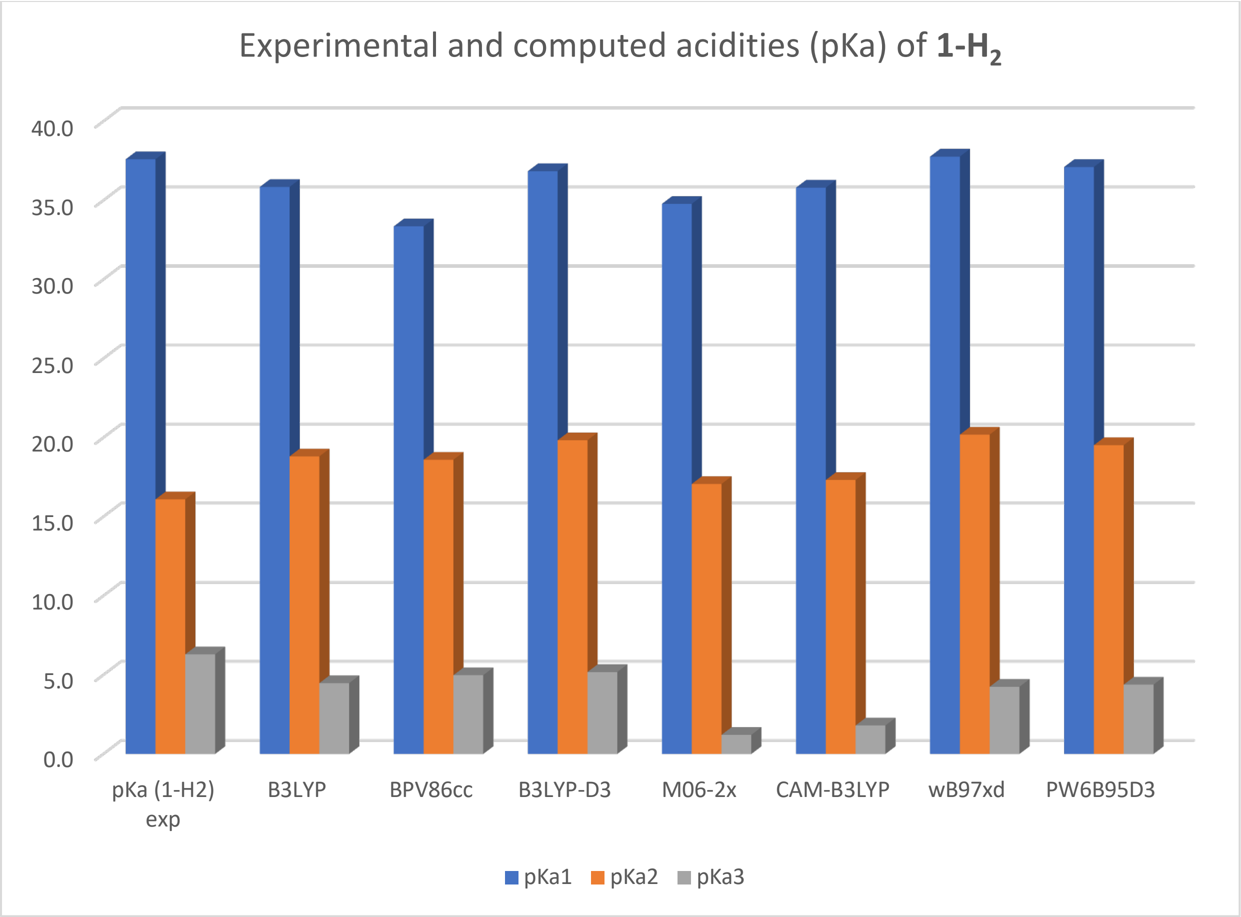

Figure S22. Comparison between methodologies for different level of computations for **1**–H_2, $\mathbf{1}^{\bullet+}$–H_2 and $\mathbf{1}^{2+}$–H_2.

Table S1. Calculated pK_a values using B3LYP/6-311++G(2d,2p)/SMD. Corrections based on experimental and calculated values for **1**–H_2, **1**$^{•+}$–H_2 and **1**$^{2+}$–H_2.

	pK_a(NH) [a]	pK_a calculated[b]	Difference	With correction[c]
1-H_2	37.6	35.9	1.8	---
1-$H_2^{•+}$	15.2	18.8	-3.6	---
1-H_2^{2+}	6.3	4.5	1.8	---
2-H_2		34.6		36.4
2-$H_2^{•+}$		19.8		16.2
2-H_2^{2+}		7.8		9.6
3-H_2		35.2		37.0
3-$H_2^{•+}$		19.0		15.4
3-H_2^{2+}		8.9		10.7

[a] Calculated in MeCN with experimental values in ref [11] and thermochemical cycle with values from ref[12]. [b]Absolute pK_a from B3LYP/6-311++G(2d,2p)/SMD using a solvent continuum of MeCN. [c] Isodesmic correction using the calculated difference for **1**–H_2, **1**$^{•+}$–H_2 and **1**$^{2+}$–H_2.

Table S2. pK_a corrections with bases.

	pK_a(NH)$_{exp}$[10]	pK_a calculated	Difference	With correction[b]
3,5-Cl_2-py		6.06		5,28
Pirazine	7.74	7.04	0,7	6,26
4-CN-py	8.50	8.73	-0,23	7,95
Py	12.53	12.88	-0,35	12,10
2,4,6-Me_3-py	15.00	16.52	-1,52	15,74
NEt_3	18.83	20.36	-1,53	19,58
DBU	24.31	25.71	-1,4	24,93
TBD	26,0	27,11	-1,11	26,33
			Avge -0.777	

[a]where py = pyridine, DBU = 1,8-Diazabicyclo(5.4.0)undec-7-ene, and TBD = 1,5,7-triazabicyclo[4.4.0]dec-5-ene. [b] Correction using the average difference of –0.777.

XYZ coordinates of structures used in DFT calculations

1-H_2

Charge = 0 Multiplicity = 1

C	-1.02097	0.73604	-0.61623
C	0.29087	1.21514	-0.61164
C	1.33882	0.49356	0.00019
C	1.02097	-0.73603	0.61623
C	-0.29087	-1.21514	0.61163
C	-1.33882	-0.49356	-0.00019
H	-1.78881	1.30903	-1.13715
H	0.51588	2.16516	-1.10503
H	1.78881	-1.30903	1.13715
H	-0.51588	-2.16516	1.10503
N	2.62374	1.06401	0.02457
H	2.64447	2.07663	-0.08978
N	-2.62373	-1.06401	-0.02457
H	-2.64447	-2.07663	0.08977
C	-3.87697	-0.44899	-0.00258
C	-5.01579	-1.25864	-0.24283
C	-4.0766	0.92187	0.28279
C	-6.30093	-0.71413	-0.20543
H	-4.87421	-2.32241	-0.45626
C	-5.37185	1.45452	0.3094
H	-3.22767	1.56807	0.50685
C	-6.49432	0.65132	0.06312
H	-7.15991	-1.36362	-0.39404
H	-5.49953	2.51642	0.53675
H	-7.49963	1.07744	0.084
C	3.87697	0.44899	0.00258
C	5.01579	1.25864	0.24283
C	4.0766	-0.92187	-0.28278
C	6.30093	0.71412	0.20543
H	4.87421	2.32241	0.45625
C	5.37184	-1.45452	-0.3094
H	3.22766	-1.56807	-0.50684
C	6.49432	-0.65133	-0.06311
H	7.15991	1.36362	0.39404
H	5.49952	-2.51642	-0.53674
H	7.49963	-1.07745	-0.08399

2-H_2

Charge = 0 Multiplicity = 1

C	-1.47409	2.0157	-0.43451
C	-0.11097	1.8086	-0.47327
C	0.45262	0.53078	-0.17628
C	-0.45262	-0.53078	0.17628

C	-1.85359	-0.29378	0.22696
C	-2.37885	0.96146	-0.08425
H	2.50159	1.11376	-0.53832
H	-1.88572	3.00118	-0.67211
H	0.55537	2.63329	-0.74147
C	1.85359	0.29378	-0.22696
C	0.11097	-1.80861	0.47327
H	-2.50159	-1.11376	0.53832
C	1.47409	-2.0157	0.43451
C	2.37885	-0.96146	0.08425
H	-0.55537	-2.63329	0.74147
H	1.88572	-3.00118	0.67211
N	3.7385	-1.29475	0.04275
H	3.92439	-2.29333	-0.0458
N	-3.7385	1.29475	-0.04275
H	-3.92439	2.29333	0.0458
C	4.88052	-0.48876	0.02724
C	6.08758	-1.06644	-0.43861
C	4.91173	0.842	0.50384
C	7.27744	-0.33565	-0.43625
H	6.07606	-2.09875	-0.80059
C	6.11149	1.56496	0.49236
H	4.0134	1.30432	0.91205
C	7.30098	0.99225	0.02075
H	8.19333	-0.80723	-0.80198
H	6.11137	2.59102	0.86982
H	8.22993	1.56642	0.01377
C	-4.88052	0.48876	-0.02724
C	-6.08758	1.06644	0.43861
C	-4.91173	-0.842	-0.50384
C	-7.27744	0.33565	0.43625
H	-6.07606	2.09875	0.8006
C	-6.11149	-1.56496	-0.49236
H	-4.0134	-1.30432	-0.91206
C	-7.30098	-0.99225	-0.02075
H	-8.19333	0.80723	0.80198
H	-6.11137	-2.59102	-0.86982
H	-8.22993	-1.56642	-0.01377

3-H$_2$
Charge = 0 Multiplicity = 1

C	2.54417	1.77444	0.27834
C	1.18549	1.46425	0.27016
C	0.72206	0.16565	-0.04677
C	1.70252	-0.79712	-0.37402
C	3.06745	-0.49798	-0.37871

C	3.51766	0.79784	-0.041
H	2.87093	2.78398	0.54441
H	0.47026	2.24356	0.54507
H	1.39403	-1.80348	-0.66866
H	3.77617	-1.26711	-0.6858
N	4.85974	1.18918	-0.05881
H	5.01052	2.19384	-0.14578
C	-0.72206	-0.16565	-0.04677
C	-1.70252	0.79711	-0.37402
C	-1.18549	-1.46425	0.27016
C	-3.06745	0.49798	-0.37871
H	-1.39403	1.80348	-0.66867
C	-2.54417	-1.77444	0.27834
H	-0.47026	-2.24356	0.54507
C	-3.51766	-0.79784	-0.041
H	-3.77617	1.26711	-0.68581
H	-2.87093	-2.78397	0.54442
N	-4.85974	-1.18918	-0.05881
H	-5.01052	-2.19384	-0.14578
C	6.03138	0.42634	-0.00578
C	7.23072	1.02781	-0.45908
C	6.09232	-0.88224	0.52529
C	8.44381	0.33878	-0.39447
H	7.1951	2.04436	-0.86161
C	7.31439	-1.56482	0.57351
H	5.19712	-1.35792	0.92595
C	8.49739	-0.96946	0.11328
H	9.35416	0.8273	-0.75167
H	7.33757	-2.57517	0.99047
H	9.44471	-1.51127	0.15473
C	-6.03138	-0.42634	-0.00577
C	-7.23072	-1.02781	-0.45908
C	-6.09232	0.88224	0.52529
C	-8.44381	-0.33879	-0.39447
H	-7.1951	-2.04436	-0.8616
C	-7.31439	1.56482	0.57351
H	-5.19712	1.35792	0.92595
C	-8.49739	0.96946	0.11328
H	-9.35416	-0.8273	-0.75167
H	-7.33758	2.57517	0.99047
H	-9.44471	1.51127	0.15473

5. Hammett type analysis

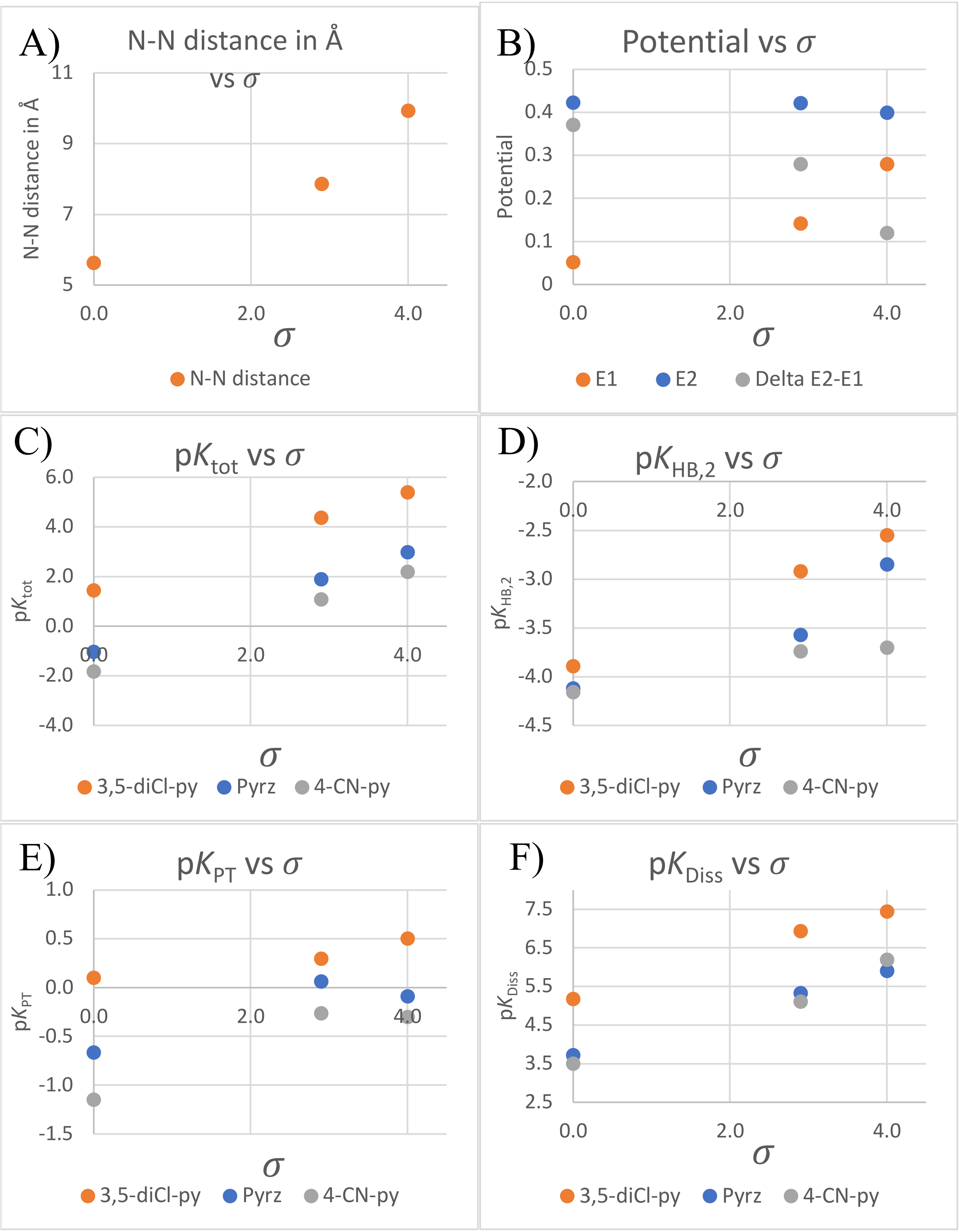

Figure S23. Hammett type analysis. The parameters plotted against the Hammett contrast are A) The nitrogen-to-nitrogen distance in Å calculated from DFT calculations, B) The electrochemical potentials $E_{1/2,1}$, $E_{1/2,2}$ and their difference ($=E_{1/2,2} - E_{1/2,1}$), C) pK_T, D) p$K_{HB,2}$, E) pK_{PT}, and F) pK_{Diss}.

In the Hammett type analysis, the A^{2+}–H_2 with different π-spacers (phenyl, naphthyl, biphenyl) act as the 'substituents' that modulate the electronic communication and electrostatic repulsion between the two redox-active nitrogen centers. The Hammett constant is calculated using the p$K_{a,2}$ of the phenyl analog as reference (σ = 0). All parameters in evaluated (Fig S23) show a strong linear correlation with σ. The strong correlation with the nitrogen-to-nitrogen distance confirms that p$K_{a,2}$ is controlled by electrostatic repulsion in the A^{2+}–H_2. The same effect accounts for the correlation with the potential difference between the first and second oxidations ($=E_{1/2,2} - E_{1/2,1}$). Small deviations from linearity occur in the correlations with p$K_{HB,2}$ and pK_{PT} with 4CN-py. We attribute the deviation to the approximation of $K_{PT} = K_{obs}/(K_{HB,2})^{1/2}$ because the experimental $K_{HB,2}$ for **1**–H_2 and **2**–H_2 corresponds to the H-bonding of 2 equivalents of base.

References

1. W. L. F. Armarego, C. Chai,. Purification of Laboratory Chemicals, Butterworth-Heinemann, ed. 7, 2013..
2. C. Costentin, M. Robert and J. M. Saveant, *Chem. Rev.*, 2010, **110**, PR1-40.
3. V. Solís, T. Iwasita and M. C. Giordano, *Journal of Electroanalytical Chemistry and Interfacial Electrochemistry*, 1976, **73**, 91-104.
4. O. V. Klymenko, D. Giovanelli, N. S. Lawrence, N. V. Rees, L. Jiang, T. G. J. Jones and R. G. Compton, *Electroanalysis*, 2003, **15**, 949-960.
5. L. A. Clare, L. E. Rojas-Sligh, S. M. Maciejewski, K. Kangas, J. E. Woods, L. J. Deiner, A. Cooksy and D. K. Smith, *J. Phys. Chem. C*, 2010, **114**, 8938-8949.
6. a) A. Matsuura, T. Nishinaga and K. Komatsu, *J. Am. Chem. Soc.*, 2000, **122**, 10007-10016; b) H. Bock, C. Arad, C. Näther and Z. Havlas, *J. Chem. Soc., Chem. Commun.*, 1995, **0**, 2393-2394.
7. C. Pratley, S. Fenner and J. A. Murphy, *Chem. Rev.*, 2022, **122**, 8181-8260.
8. M. J. Frisch, G. W. Trucks, H. B. Schlegel, G. E. Scuseria, M. A. Robb, J. R. Cheeseman, G. Scalmani, V. Barone, G. A. Petersson, H. Nakatsuji, X. Li, M. Caricato, A. Marenich, J. Bloino, B. G. Janesko, R. Gomperts, B. Mennucci, H. P. Hratchian, J. V. Ortiz, A. F. Izmaylov, J. L. Sonnenberg, D. Williams-Young, F. Ding, F. Lipparini, F. Egidi, J. Goings, B. Peng, A. Petrone, T. Henderson, D. Ranasinghe, V. G. Zakrzewski, J. Gao, N. Rega, G. Zheng, W. Liang, M. Hada, M. Ehara, K. Toyota, R. Fukuda, J. Hasegawa, M. Ishida, T. Nakajima, Y. Honda, O. Kitao, H. Nakai, T. Vreven, K. Throssell, J. A. Montgomery, Jr., J. E. Peralta, F. Ogliaro, M. Bearpark, J. J. Heyd, E. Brothers, K. N. Kudin, V. N. Staroverov, T. Keith, R. Kobayashi, J. Normand, K. Raghavachari, A. Rendell, J. C. Burant, S. S. Iyengar, J. Tomasi, M. Cossi, J. M. Millam, M. Klene, C. Adamo, R. Cammi, J. W. Ochterski, R. L. Martin, K. Morokuma, O. Farkas, J. B. Foresman, D. J. Fox, Gaussian 09, Revision A.02, Gaussian, Inc., Wallingford CT, 2016.
9. a) J. Tirado-Rives and W. L. Jorgensen, *J. Chem. Theory Comput.*, 2008, **4**, 297-306; b) M. T. Huynh, C. W. Anson, A. C. Cavell, S. S. Stahl and S. Hammes-Schiffer, *J. Am. Chem. Soc.*, 2016, **138**, 15903-15910.
10. S. Tshepelevitsh, A. Kütt, M. Lõkov, I. Kaljurand, J. Saame, A. Heering, P. G. Plieger, R. Vianello and I. Leito, *Eur. J. Org. Chem.*, 2019, **2019**, 6735-6748.
11. a) X.-M. Zhang and F. G. Bordwell, *J. Org. Chem.*, 2002, **59**, 2809-2812; b) F. G. Bordwell, X. M. Zhang and J. P. Cheng, *J. Org. Chem.*, 1993, **58**, 6410-6416.
12. R. G. Agarwal, S. C. Coste, B. D. Groff, A. M. Heuer, H. Noh, G. A. Parada, C. F. Wise, E. M. Nichols, J. J. Warren and J. M. Mayer, *Chem. Rev.*, 2021, **122**, 1-49.
13. a) A. D. Becke, *J. Chem. Phys.*, 1993, **98**, 5648-5652; b) C. Lee, W. Yang and R. G. Parr, *Phys Rev B Condens Matter*, 1988, **37**, 785-789.
14. a) A. D. McLean and G. S. Chandler, *J. Chem. Phys.*, 1980, **72**, 5639-5648; b) R. Krishnan, J. S. Binkley, R. Seeger and J. A. Pople, *J. Chem. Phys.*, 1980, **72**, 650-654.
15. A. V. Marenich, C. J. Cramer and D. G. Truhlar, *J. Phys. Chem. B*, 2009, **113**, 6378-6396.
16. S. J. Konezny, M. D. Doherty, O. R. Luca, R. H. Crabtree, G. L. Soloveichik and V. S. Batista, *J. Phys. Chem. C*, 2012, **116**, 6349-6356.
17. J. Ho and M. L. Coote, *Theoretical Chemistry Accounts*, 2010, **125**, 3-21.
18. E. Rossini and E. W. Knapp, *J. Comput. Chem.*, 2016, **37**, 2163-2164.
